# Tumor-targeted multifunctional extracellular vesicles as drug carriers for lung cancer therapy

**DOI:** 10.20517/evcna.2025.39

**Published:** 2025-12-24

**Authors:** Narsireddy Amreddy, Akhil Srivastava, Natascha Riedinger, Murali Ragothaman, Yan D. Zhao, Hariprasad Gali, Anupama Munshi, Rajagopal Ramesh

**Affiliations:** ^1^Department of Pathology, University of Oklahoma Health Sciences Center, Oklahoma City, OK 73104, USA.; ^2^OU Health Stephenson Cancer Center, University of Oklahoma Health Sciences Center, Oklahoma City, OK 73104, USA.; ^3^Boone Pickens School of Geology, Oklahoma State University, Stillwater, OK 74078, USA.; ^4^Department of Biostatistics and Epidemiology, University of Oklahoma Health Sciences Center, Oklahoma City, OK 73104, USA.; ^5^Department of Pharmaceutical Sciences, University of Oklahoma Health Sciences Center, Oklahoma City, OK 73104, USA.; ^6^Department of Radiation Oncology, University of Oklahoma Health Sciences Center, Oklahoma City, OK 73104, USA.; ^†^Current Address: Cytovance Biologics, Oklahoma City, OK 73104, USA.; ^‡^Current Address: Department of Pathology and Anatomical Sciences, University of Missouri School of Medicine, Columbia, MO 65212, USA.

**Keywords:** Extracellular vesicles, lung cancer, drug delivery, chemotherapy, tumor targeting

## Abstract

**Aim:** Chemotherapy continues to be the frontline treatment for lung cancer patients. However, treatment-related toxicity and off-target effects limit the use of chemotherapy. Therefore, improvements in delivering chemotherapeutics with reduced toxicity to normal tissues are needed. In the present study, we combined nanotechnology with extracellular vesicle (EV) technology to produce tumor-targeted multifunctional EVs (tt-Mfn-EVs) as drug carriers for cancer therapy.

**Methods:** The tt-Mfn-EVs were formulated by exogenously loading EVs with gold nanoparticles conjugated to cisplatin (CDDP) via pH-sensitive coordination ester linkage. Attached to the outer surface of drug-loaded EVs is the transferrin ligand for targeting transferrin receptor (TfR) overexpressing lung cancer cells.

**Results:** The tt-Mfn-EVs were 138.2 nm in size and exhibited greater drug release kinetics at pH 5.5 compared to pH 7.2. They significantly reduced cell viability of A_549_ (TfR high) lung cancer cells compared to HCC_827_ (TfR low) cells and non-targeted EVs. Tt-Mfn-EVs also induced higher levels of apoptosis and DNA damage in A_549_ and HCC_827_ cells compared to control groups. Finally, tt-Mfn-EV-mediated cytotoxicity was minimal in normal human lung fibroblast (MRC-_9_) and human embryonic kidney 293 (HEK_293_) cells compared to free CDDP.

**Conclusion:** Our study showed that tt-Mfn-EVs exerted selective and enhanced tumor-targeted cell killing *in vitro*, providing an opportunity for developing EV-based drug carriers for cancer therapy.

## INTRODUCTION

Chemotherapy is the frontline treatment for the majority of cancers, including lung cancer^[1-4]^. Treatment-related non-specific toxicity, however, limits the dosage and continued use of chemotherapy to achieve therapeutic efficacy^[5,6]^. Additionally, a large number of anticancer drugs are hydrophobic and poorly soluble, requiring their loading or conjugation to drug carriers for application in cancer treatment^[7,8]^. To overcome the solubility issue of the anticancer drugs, several biological and non-biological-based drug carriers have been tested for cancer therapy^[9,10]^. In more recent years, extracellular vesicles (EVs) have gained interest in their use as drug delivery carriers^[11-13]^. EVs are ubiquitously produced by all cell types and have several advantages, including their small size, ability to freely circulate and traverse across cell membranes, and are non-immunogenic^[14-16]^. The added advantage of EVs as drug carriers is their ability to escape the lysosome/endosomal trapping, thereby enabling efficient drug delivery^[17,18]^. Due to these unique properties, EVs as drug delivery vehicles are being tested for a wide variety of human diseases, including cancer.

For cancer treatment, several approaches for loading chemotherapeutics into EVs have been used^[19,20]^. Each of the drug loading methods, while having advantages, also suffers from disadvantages. For example, physical methods such as electroporation, sonication, and eddy oscillation can disrupt the membrane structure of the EVs, leading to rearrangement and regeneration of different particle sizes. Similarly, simple incubation of free drug, while efficient in the loading of the drug into EVs, suffers from rapid and uncontrolled drug release that could contribute to non-specific delivery and toxicity. Therefore, combining the simple incubation technique for drug loading with a controlled drug release strategy will be advantageous for the application of EV-based cancer therapy^[21,22]^.

Herein, we combined nanotechnology with EV technology for producing tumor-targeted multifunctional EVs (tt-Mfn-EVs) as drug delivery vehicles for cancer therapy. In this study, metallic gold nanoparticles (GNPs) were used for conjugating chemodrug. The rationale for using GNPs is that they are small, biocompatible, and offer a large surface area for conjugating therapeutics^[23-25]^. Additionally, GNPs can be used for optical imaging and thermal therapy^[26]^. *In vivo*, use of GNPs in their free form results in rapid clearance and reduced efficacy due to aggregation and protein corona formation^[27]^. However, loading of GNPs into EVs will not only reduce their clearance from circulation but also allow incorporating anticancer drugs which, when conjugated using specific linkers, can selectively release the drug under a defined tumor microenvironment^[28]^. Thus, GNP-drug-loaded EVs can selectively release the drug for achieving tumor-selective cell killing. To further enhance tumor selectivity, these EVs can be surface modified with ligand moieties targeted towards cell surface receptors that are overexpressed in cancer cells compared to normal cells^[29-31]^. This approach will enhance anticancer activity with reduced cytotoxicity to normal cells, a feature highly preferred in cancer treatment and of clinical relevance. To achieve these objectives, we developed and tested tt-Mfn-EVs using lung cancer as a model.

The tt-Mfn-EV is formulated by loading cisplatin (CDDP) conjugated to GNPs via a pH-sensitive coordinate ester linker into EVs derived from normal human lung fibroblast (MRC-_9_) cells. The final formulation was surface decorated with transferrin (Tf) ligand targeting the transferrin receptor (TfR). The rationale for selecting CDDP and Tf is that CDDP is the frontline chemodrug for lung cancer treatment, and TfR is overexpressed in lung cancer compared to normal cells and tissues^[32,33]^. The tt-Mfn-EVs were subjected to physico-chemical characterization followed by molecular and efficacy studies against human lung cancer cells and normal cells *in vitro*. Our study results showed that tt-Mfn-EVs exerted selective and significant cytotoxicity towards tumor cells and spared normal cells with minimal toxicity *in vitro.* Additionally, our study provides evidence for developing EV-based drug carriers for cancer therapy. However, testing of tt-Mfn-EVs in *in vivo* lung tumor models in the future is required prior to advancing to clinical translation.

## METHODS

### Materials

The materials used in the study were purchased from commercial vendors and included the following: Methoxypolyethylene glycol thiol (HS-PEG-OMe) (8,000 Da), human transferrin (hTf), iminothiolane, trisodium citrate, gold chloride, CDDP, silver nitrate, orthophenylene diamine (OPDA), isopropyl ethylamine, N,N-dimethylformamide (DMF) and ethylenediaminetetraacetic acid (EDTA) were all purchased from Sigma Chemicals (‎St. Louis, MO, USA). HS-PEG-COOH [3,400 molecular weight; Nanocs, New York, NY, USA]; maleimide-PEG-1,2-distearoyl-sn-glycero-3-phosphoethanolamine (DSPE) lipid (2,941 MW; Avanti polar lipids, Alabaster, AL, USA); Analytical reagent (AR) grade formic acid (FA), 4, 6-diamidino-2-phenylindole (DAPI) and Pierce^TM^ bicinchoninic acid (BCA) protein assay (Thermo Scientific, Massachusetts, MA, USA); Exo-kit (System Biosciences, Palo Alto, CA, USA); SephadexG-25 (PD10; GE, PA, USA); bovine serum albumin (BSA; KSE Scientific, Durham, NC, USA); trypan blue (Lonza, MD, USA); polyvinylidene fluoride-membrane (Millipore, Billerica, MA, USA); liquid chromatography/mass spectrometry (LC/MS) CHROMASOLV® grade acetonitrile (ACN) and water, dithiothreitol (DTT), iodoacetamide (IAA), and urea (Sigma-Aldrich, St. Louis, MO, USA). The packing materials for packing C18 (Jupiter particles, 5 μm diameter, 300 Å pore size) columns were purchased from Phenomenex (Torrance, CA, USA); ExoGlow^TM^-Membrane EV Labeling Kit (System Biosciences Inc, CA, USA).

### Cell lines

Non-small cell lung cancer (NSCLC; A_549_, HCC827 and H1299), normal MRC-_9_ and human embryonic kidney 293 (HEK_293_) cell lines were obtained from American Type Culture Collection (ATCC; Manassas, VA, USA). A_549_, HCC827, H1299, and HEK_293_ cell lines were maintained in Roswell Park Memorial Institute (RPMI) 1640 media (GIBCO BRL Life Technologies, NY, USA) supplemented with 10% of exosome-depleted fetal bovine serum (FBS) (System Biosciences, Palo Alto, CA, USA) and 1% penicillin-streptomycin. MRC-_9_ cells were cultured in Eagle’s Minimum Essential Medium (EMEM) media (GIBCO BRL Life Technologies, NY, USA) with 10% exosome-depleted FBS and 1% penicillin-streptomycin. All cells were sub-cultured at 70% of confluence and tested for mycoplasma prior to use in the studies.

### EVs isolation and purification

EVs derived from MRC-_9_ cells were used in the present study. The rationale for using EVs from MRC-_9_ cells was multifactorial and included their physiological relevance to a lung tissue model and the fact that EV yield from MRC-_9_ cells was ten-fold higher than that from human telomerase reverse transcriptase (hTERT)-immortalized mesenchymal stem cells (MSCs) and hTERT-immortalized lung fibroblasts, limiting the utility of the latter for downstream applications. Hence, EVs from MRC-_9_ were isolated and purified as described below and in the present study.

EVs from MRC-_9_ cell culture supernatant were isolated using high-speed differential centrifugation and the ultracentrifugation method. Briefly, cell culture supernatants were collected and centrifuged at 10,000 × *g* for 30 min at 4 °C to remove cell debris. The resulting supernatant was filtered through a 200 nm pore filter and collected in ultracentrifuge tubes, followed by ultracentrifugation at 100,000 × *g* for 120 min at 4 °C. The supernatant was discarded, and the EV pellet was resuspended in 200 µL 1× PBS and stored at -80 °C until further use.

### GNP synthesis

GNPs were synthesized by the citrate-stabilized method. Briefly, 20 mL of 1 mM HAuCl_4_ solution was vigorously stirred at 80 °C until boiling, after which 3 mL of 1% trisodium citrate solution was gently added. Stirring was continued until the solution turned wine red in color. The solution was then left to cool at room temperature to allow the GNPs to stabilize for subsequent use in the studies.

### GNP-CDDP synthesis

Prior to conjugating CDDP to GNPs, the CDDP was converted into hydrated CDDP by the silver nitrate method^[34]^. Briefly, 150 mg of CDDP was dispersed in 2.5 mL of Milli-Q water and added to a tube containing 170 mg of silver nitrate (AgNO_3_) dissolved in 2.5 mL of Milli-Q water. The solution was mixed and heated at 50 °C for 1 h followed by centrifugation at 13,500 rpm for 15 min. The supernatant containing the hydrated form of CDDP was collected and filtered by a 0.22 μm Millex® Syringe filter (EMD Millipore, Cork, Ireland) to remove any residual contaminants. The CDDP concentration in the final hydrated CDDP solution was determined using the OPDA assay. The final CDDP solution was stored at 4 °C and used in the study.

For conjugating hydrated CDDP with GNPs, the surface of GNPs was modified with PEG linkers. Briefly, 20 mL of citrate-stabilized GNPs was mixed with 2 mL of 1 mM HS-PEG-OMe (8,000 MW) and incubated overnight at room temperature to prevent GNP aggregation. The mixture was then centrifuged to remove any unbound HS-PEG-OMe followed by the addition of 1 mL of 1 mM HS-PEG-COOH linker to the dispersed GNP solution with continuous stirring for 24 h at room temperature. Any unconjugated HS-PEG-COOH in the preparation was removed by centrifugation at 15,000 rpm for 15 min, after which the supernatant was discarded. The GNP-PEG-COOH pellet was dispersed in 5 mL of PBS and 100 µL of 0.1 mM isopropyl ethylamine base was added to activate the –COOH functional groups and stirred for 1 h at room temperature. The unbound isopropyl ethylamine was removed by centrifugation at 15,000 rpm for 15 min. To the activated GNP-PEG-COOH solution, 15 mg of hydrated CDDP was added and the solution was mixed by stirring for 24 h at room temperature followed by centrifugation at 13,500 rpm for 15 min to remove any unbound CDDP. The amount of CDDP bound in the GNP-PEG-COO-CDDP complex was estimated by the OPDA assay. Hereafter, this GNP-PEG-COO-CDDP complex was referred to as GNP-CDDP.

### EV loading of GNP-CDDP

Briefly, GNP-CDDP was loaded onto MRC-_9_-derived EVs by mixing 1 mg equivalent of CDDP contained in GNP-CDDP with 500 µg protein equivalent EVs, followed by incubation at 37 °C for 2 h with mild stirring. The GNP-CDDP-loaded EVs were subsequently purified using the ExoQuick for Tissue Culture (ExoQuick-TC) reagent kit (System Biosciences, Palo Alto, CA, USA). The amount of CDDP present in the GNP-CDDP-loaded EVs was determined by the OPDA assay.

### Conjugation of Tf-DSPE lipid and insertion into EVs

hTf (76-81kDa MW) was first converted into thiolate Tf by treating with the iminothiolane reagent. Briefly, 6.5 mg of Tf was dissolved in 2 mL of 0.1 M Na_2_HPO_4_ containing 0.1 M EDTA at pH 8.0, after which 0.25 mg of iminothiolane was added and the mixture was stirred for 3 h at 4 °C to form the Tf-iminothiolane (Tf-SH) solution. Any unbound iminothiolane was removed using a PD10 desalting column with 0.15 M NaCl solution used as column equilibrating buffer and 0.1 M Na_2_HPO_4_ with 0.1 M EDTA at pH 7.1 as eluent buffer. Then, Tf concentration was estimated using the BCA protein assay and 2.7 mg of purified Tf-SH was mixed with 1 mg of maleimide-PEG-DSPE lipid (2,941 MW) and allowed to react overnight at 4 °C. The resulting Tf-DSPE lipid was purified by centrifugation at 15,000 rpm for 15 min and the Tf concentration in the Tf-DSPE lipid was estimated using the BCA protein assay kit.

For inserting the Tf-DSPE lipid into EVs, 100 µg protein equivalent of GNP-CDDP-loaded EVs were dispersed in 100 µL PBS (pH 7.4). To the EVs, 4 µg of transferrin equivalent in the Tf-DSPE lipid complex was added and incubated at 37 °C for 1 h with mild stirring. The Tf-DSPE conjugated EVs were purified using the Exo-Quick-TC reagent kit (System Biosciences) and used in the study. For estimation of Tf by mass spectrometry, Tf-DSPE was added to empty EVs (without GNP-CDDP) and analyzed.

### Characterization of EVs and tt-Mfn-EVs

The concentration and size of the EVs were analyzed by Nanotracker analysis (NanoSight NS300, Malvern Panalytical Inc., MA, USA). The number and size of EVs were analyzed by capturing three video cycles for 60 s and using the NanoSight software. For determining the surface charge, EVs and GNP-CDDP complexes were dispersed in PBS and measured with the Zeta PALS instrument (Brookhaven Instruments, Holtsville, NY, USA) at pH 7.4. The size and shape of the EVs, GNPs, and GNP-loaded EVs were determined by transmission electron microscopy (TEM, 80 kV, Hitachi 7600, Tokyo, Japan). The samples were dispersed in PBS, and 20 µL of the dispersed samples were placed on copper grids and allowed to adsorb for 60 min. Excess sample solution was removed from the grids using filter paper and then stained with 1% phosphotungstic acid (PTA). The stained samples were then subjected to TEM, and photographs were captured. The absorption spectra of GNPs and GNP conjugations were measured using ultraviolet (UV)-visible (Vis) spectra of SpectraMax iD3 and iD5 Hybrid Multi-Mode Microplate Reader (Molecular Device, CA, USA). The Fourier transform infrared (FTIR) spectroscopy was used to record vibrational spectra in the range of 950-4,000 cm^-1^ with the Bruker Tensor II FTIR Confocheck (Billerica, MA, USA) BioATRCell II mode.

### OPDA assay

The drug conjugation efficiency and drug loading efficiency were determined by the OPDA colorimetric assay^[35]^. Briefly, 700 µg of OPDA was added to CDDP-containing samples and to standards in the presence of 500 µL of DMF and incubated at 95 °C for 30 min. On completion of the reaction indicated by change in color from colorless to blue, the optical density of the samples was measured at 704 nm using a Denovix DS-11 spectrophotometer (Wilmington, DE, USA). CDDP concentration in the test samples was plotted against the standard curve and the CDDP conjugation and loading efficiencies were expressed as a percentage using: % Conjugation or loading efficiency = (Initially added CDDP weight - weight of CDDP left in the supernatant) / Initial weight of CDDP added × 100.

### Drug release and confirmation of pH linkage

To confirm the CDDP conjugation with GNPs through pH-sensitive linkage and to study the CDDP release properties in acidic and neutral pH environments, two sets of buffers - acetate buffer (ABS; pH 5.5) and phosphate buffer (PBS; pH 7.4) - were used. Briefly, 50 µL of GNP-CDDP and EV-GNP-CDDP complexes dispersed in 150 µL of ABS and PBS solutions were loaded in mini dialysis tubes (Thermo Scientific, MA, USA) and placed inside 1.5 mL Eppendorf tubes containing 1.2 mL of PBS and ABS. The Eppendorf tubes were placed on a magnetic stirrer and stirred at 220 rpm at 37 °C. At defined time intervals, 200 µL of buffer solution was drawn from each of the sample containing tubes and replenished with an equal volume of fresh buffer in the respective tubes. The CDDP concentration in the samples collected at different time points was estimated using the OPDA assay and the drug release rate was calculated by comparing against drug concentration standards.

### Dot blot and mass spectrometry

The Tf loading onto the EVs was determined by dot blot assay. Tf conjugated DSPE lipid (Tf-DSPE lipid) was used as a positive control in the assay and non-Tf conjugated EVs were used as a negative control. Tf conjugation in the EVs was detected using cluster of differentiation (CD)71 mouse monoclonal primary antibody (Cell Signaling, 1:200 dilution with 5% BSA) and an appropriate secondary anti-mouse antibody (1:1,000).

Tf conjugation on EVs by mass spectrometry was also determined by the bottom-up liquid chromatography–tandem mass spectrometry (LC-MS/MS) approach. Briefly, Tf labeled EVs were vacuum dried and reconstituted in a solution containing 25 mM ammonium bicarbonate (ABC) and 6 M urea, followed by treatment with DTT (5 mM final concentration) and IAA (10 mM final concentration). The samples were subjected to tryptic digestion by the addition of trypsin with a protein to enzyme ratio of 50:1, and the enzymatic digestion was performed overnight at 37 °C. The digested fractions were desalted and loaded onto an in-house packed C18 column (5 μm, 75 μm × 15 cm) for the bottom-up study. The mobile phases were 0.1% FA in water (mobile phase A, MPA) and 0.1% FA in ACN (mobile phase B, MPB). The gradient was from 3% MPB to 35% MPB over 60 min. The reversed-phase liquid chromatography (RPLC) column was coupled directly to an LTQ Orbitrap Velos Pro mass spectrometer (Thermo Fisher Scientific, Bremen, Germany) through a home-made nano-electrospray ionization (nano-ESI) source. The obtained LC-MS/MS spectrum provided information regarding the sequence of Tf and their quantitative values. EVs not labeled with Tf served as negative control.

### Subcellular compartment pH measurement studies

The pH in the subcellular compartment was measured in tumor (A_549_, HCC827), normal lung fibroblast (MRC-_9_), and human embryonic kidney (HEK_293_) cells using the pHrodo^TM^ Red AM Intracellular pH Indicator (Thermo Fisher Scientific, Eugene, OR, USA) per manufacturer’s recommendation. Briefly, 1 × 10^4^ cells were seeded in each well of a 96-well black plate. At 24 h after seeding, the cells were washed with 1× PBS and incubated for 30 min at 37 °C with 100 µL of pHrodo^TM^ Red AM (1 µL of stock in 1 mL of HEPES pH 7.4 buffer), and each cell line was tested in triplicate. After 30 min of incubation, one set of cells for each cell line was used for making a pH standard curve and another set was used for testing pH values. For the standard curve, cells were incubated at 37 °C in 100 µL of pH 4.5 buffer which contains 10 µM of Nigericin and Valinomycin for 5 min and fluorescence readings were taken at 580 nm/540 nm emission and excitation wavelength, respectively, using SpectraMax iD3 and iD5 Hybrid Multi-Mode Microplate Reader (Molecular Devices, CA, USA). The cells were subsequently washed and incubated sequentially with pH 5.5, pH 6.5 and pH 7.5 buffers and the fluorescence reading was obtained at 580 nm/540 nm wavelength. To obtain the pH measurement in test samples, cells were washed after 30 min incubation with PBS and fluorescence values were measured using the Multi-Mode Microplate Reader. The pH values of the test samples were determined against the standard using the linear curve equation Y = MX, where Y = fluorescence intensity of the test cell line in pHrodo^TM^ Red; M = slope of the standard curve; and X = subcellular compartment pH of the test cell line.

### Cell uptake studies

EVs derived from MRC-_9_ cells were labeled with a fluorescent dye using the ExoGlow^TM^-Membrane EV Labeling Kit (System Biosciences Inc, CA). Labeling of the EVs followed the protocol recommended by the manufacturer and unbound dye was removed to minimize non-specific labeling. The cells (A_549_, MRC-_9_) were seeded on cover slips (5 × 10^4^) or six-well plates (1 × 10^5^) and were treated with unlabeled EVs (100 µg protein equivalent), fluorescently labeled-non-targeted drug-loaded EVs (EV-GNP-CDDP), and tt-Mfn-EVs. Cells that did not receive any treatment served as control. At 24 h after treatment, cells grown on cover slips were subjected to fluorescence microscopy and the cells grown on six-well plates were subjected to fluorescence measurement. The fluorescence intensity measured was normalized to 10,000 cells for each treatment group, and the differences in the fluorescence intensity as a measure of cell uptake were assessed for statistical significance.

To exclude the possibility that the observed fluorescence was due to non-specific dye uptake, we applied the ExoGlow^TM^-Protein EV Labeling Kit (System Biosciences Inc.) for assessing cell uptake of EVs. The kit enables covalent fluorescent labeling of internal EV proteins thereby reducing background signal and providing accurate assessment of cell uptake and internalization of EVs. A_549_ cells seeded on coverslips (5 × 10^4^ cells per well) and placed in six-well plates were treated with EVs labeled with ExoGlow^TM^ Protein Labeling kit. Cells that were treated with free dye and cells receiving no treatment served as controls. After 24 h, the cells grown on coverslips were subjected to fluorescence microscopy.

### Inductively coupled plasma mass spectrometry analysis

Gold (Au) and platinum (Pt) concentration in the EV-treated cells was determined by inductively coupled plasma-mass spectrometry (ICP-MS). A_549_ cells seeded in six-well plates (1 × 10^5^/well) were treated with free CDDP, GNP-CDDP, EV-GNP-CDDP (non-targeted drug-loaded EVs) and EV-GNP-CDDP-Tf (tt-Mfn-EVs). In each treatment group, equal concentrations of CDDP (30 µg/well) and Tf (4 µg/well) were maintained. After 24 h of treatment, each group of cells was washed with PBS and collected by trypsinization. The cells were dissolved in 5% aqua regia to leach out metal ions, and the Au and Pt concentrations were determined using ICP-MS (iCAP^TM^ Qc, Thermo Scientific, Waltham, MA, USA) with appropriate dilutions. The numbers of Au and Pt atoms were calculated using Avogadro’s number, and the number of EVs that entered the cells was estimated by comparing these values with the number of Au and Pt atoms initially added to the EVs.

### Fluorescence microscopy

A_549_ cells (5 × 10^4^) were seeded on coverslips placed in a six-well plate. After 12 h of incubation, the culture medium was aspirated, washed with PBS twice, and fresh EV-free serum-containing medium was added and treated with fluorescently labeled EV-GNP-CDDP and tt-Mfn-EVs. Cells that were treated with unlabeled EVs and cells receiving no treatment served as controls. At 24 h after treatment, the cells were washed three times with ice-cold PBS, each wash lasting 5 min, and then fixed with 4% paraformaldehyde (PFA) for 20 min. Following an additional wash with ice-cold PBS, the cells were counterstained with DAPI for 5 min to stain nuclei. The cells were washed in ice-cold PBS three times, and the cover slip was removed and mounted on a glass slide with gold anti-fade mounting reagent (Molecular Probes, Life Technologies, USA). The fluorescence images were acquired using a Nikon epifluorescence microscope (Nikon Instruments, NY, USA).

For MRC-_9_ cells, they were treated with fluorescently labeled EV-GNP-CDDP and tt-Mfn-EVs and compared to untreated cells. All other experimental conditions and evaluation time were identical to those described above for A_549_ cells.

### Cell viability assay

The cell viability studies were performed as previously described^[36]^. Briefly, A_549_, HCC827, and MRC-_9_ were seeded in 100 mm dish (4 × 10^5^ cells/dish). At 24 h after seeding, the culture medium was replaced with serum-free medium for 1 h and then treated with free CDDP, GNP-CDDP, EV-GNP-CDDP (non-targeted drug-loaded EVs) and EV-GNP-CDDP-Tf (tt-Mfn-EVs). After 6 h of treatment, the culture media were replaced with 5% serum-containing media in the respective wells. In each treatment group, equal concentrations of CDDP (150 µg/well) and Tf (20 µg/well) were maintained. Untreated cells served as the control. The cells were harvested at various times after treatment, and the number of viable cells in each treatment group was counted. The results were expressed as percent viable cells over untreated control cells for each cell line.

For HEK_293_ cells, they were seeded at 1 × 10^5^ cells/dish and treated as described above. The only difference is that these cells were treated with 30 µg of CDDP and 4 µg of Tf equivalent concentration in the treatment groups. Untreated cells served as control. All other experimental conditions remained the same as described above for lung tumor cells and MRC-_9_ cells.

### TfR blocking study

A_549_ cells suspended in 10% FBS-containing medium were seeded in a 100 mm dish (4 × 10^5^ cells/dish). After 24 h of seeding, the culture medium was replaced with serum-free culture medium, and incubation continued for 1 h. Then, in two of the dishes, 100 and 200 µg/mL hTf were added, followed by an additional one-hour incubation. Subsequently, to these two dishes, tt-Mfn-EVs were added. To the remaining dishes, EV-GNP-CDDP (non-targeted drug-loaded EVs) and tt-Mfn-EVs alone were treated on the cells without hTf. Cells that did not receive any treatment served as control. In all the dishes, EVs containing equivalent concentrations of CDDP (150 µg) and Tf (20 µg) were added. After 24 h of treatment, the cells were harvested by trypsinization, with an aliquot used for cell viability assays and the remaining cells used for molecular analysis by western blotting.

### Fluorescence measurements

A_549_ cells (1 × 10^5^) were seeded in six-well plates. After 8-12 h of incubation, the culture medium was aspirated, washed with PBS twice, and fresh EV-free serum-containing medium was added and treated with fluorescently labeled EV-GNP-CDDP and tt-Mfn-EVs (7.5 × 10^10^ equivalent to 100 μg protein). Cells that were treated with unlabeled EVs and cells receiving no treatment served as controls. After 24 h of treatment, cells were harvested and washed once with ice-cold PBS and resuspended in 1 mL of PBS. An aliquot (200 μL) of the cells was added in duplicate wells in a black six-well plate, and the fluorescence intensity was measured at 465 nm excitation/635 nm emission using SpectraMax ID3 plate reader (Molecular Devices). Fluorescence intensity measured was normalized to 10,000 cells for each group. The results obtained were subjected to statistical analysis with significance set at < 0.05.

For MRC-_9_ cells, cells were treated with fluorescently labeled EV-GNP-CDDP and tt-Mfn-EVs and compared to untreated cells. All other experimental conditions and evaluation time were identical to those described above for measuring fluorescence intensity in A_549_ cells.

### Labeling of liposomes

Tf conjugated DOTAP (1,2-dioleoyl-3-trimethylammonium-propane): Cholesterol liposomes were synthesized as described previously^[37]^. The Tf-liposomes were labeled with ExoGlowTM-Membrane EV Labeling dye following the manufacturer’s protocol with slight modification. After adding the dye and incubating it for 45 min at room temperature, the liposomes were centrifuged at 25,000 × *g* for 30 min, followed by washing with 1× PBS. The labeled liposomes were resuspended in PBS and the number of liposomes was determined by the Nanotracker Analysis System (NanoSight NS300). The labeled liposomes were subsequently used for cell uptake studies and compared to labeled EVs.

### Comparison of cell uptake of liposomes with EVs

A_549_ cells seeded in cover slips (5 × 10^4^) and in six-well plates (1 × 10^5^) were treated with an equivalent amount of fluorescently labeled Tf-Liposomes and Tf-EVs (7.5 × 10^10^ equivalent to 100 µg protein) and subjected to fluorescence microscopy and fluorescence measurement as described in the earlier section. Unlabeled EVs served as control.

### Western blot analysis

Total protein concentration in cell lysates from A_549_, HCC827, MRC-_9_ and HEK_293_ and from MRC-_9_-derived EVs was determined using the BCA assay (Pierce^TM^ BCA Protein Assay Kit, Thermo Fisher Scientific) according to the manufacturer’s instructions. EV-specific markers were determined by western blot analysis. Primary antibodies used included CD63, tumor susceptibility gene (TSG)101, and Annexin A1 (1:1,000; Abcam, Waltham, MA, USA); CD81, Alix, argonaute 2 (AGO2), Calnexin, and golgi matrix protein 130 (GM130) (1:1,000; Cell Signaling Technology, Beverly, MA, USA); and CD9 (1:1,000; Proteintech, Rosemont, IL, USA).

For analysis of TfR protein expression in the human lung cancer and normal cell lines, total cell lysates were probed for TfR using anti-TfR antibody (CD71; 1:1,000; Abcam). For analysis of apoptotic and DNA damage markers in therapeutic efficacy studies, total cell lysates were probed for poly (ADP-ribose) polymerase (PARP), Caspase-9 and gamma Histone 2A family member X (γH_2_AX) (1:1,000 dilution; Cell Signaling Technology Inc.). Beta actin (1:2,000; Sigma Chemicals) was used as an internal control in the western blot analyses. Proteins were detected with appropriate horse radish peroxidase (HRP)-conjugated secondary antibody and images captured using the chemiluminescence imaging system (Syngene, Frederick, MD, USA). The captured images were quantified with Image Quant software (Biochemlab Solutions, San Francisco, CA, USA).

### Annexin V/PI staining

Apoptotic cell death of lung cancer cell lines was determined with Alexa Fluor-488-Annexin V/propidium iodide (PI) (Thermo Fisher Scientific) staining kit. A_549_ and HCC827 cells seeded in six-well plates (8 × 10^4^/well) were treated with free CDDP, GNP-CDDP, EV-GNP-CDDP (non-targeted drug-loaded EVs) and tt-Mfn-EVs. All treatments contained equal concentrations of CDDP (30 µg) and Tf (4 µg). Untreated cells served as control groups. After 24 and 48 h of treatment, the cells were harvested and stained with 5 μL of Alexa Fluor-488-Annexin V and 1 μL of PI (1 mg/mL) solution for 15 min at room temperature as per the manufacturer’s protocol. The percentage of apoptotic cells was calculated using a fluorescence activated cell sorting (FACS) Calibur flow cytometer using the Cell Quest software (BD Biosciences).

### Statistics

All the data were expressed as mean ± standard deviation, and all the experiments were conducted at least three separate times. Statistical analyses were performed for variables of percent cell viability, intracellular pH measurement, and western blot quantification compared among treatment and untreated groups using an unpaired Student’s *t*-test. A *P*-value of less than 0.05 was considered statistically significant, and statistical analysis was performed using the GraphPad Prism software version 10.5.

## RESULTS

### Bio-physical characterization of EVs

**Figure 1:**
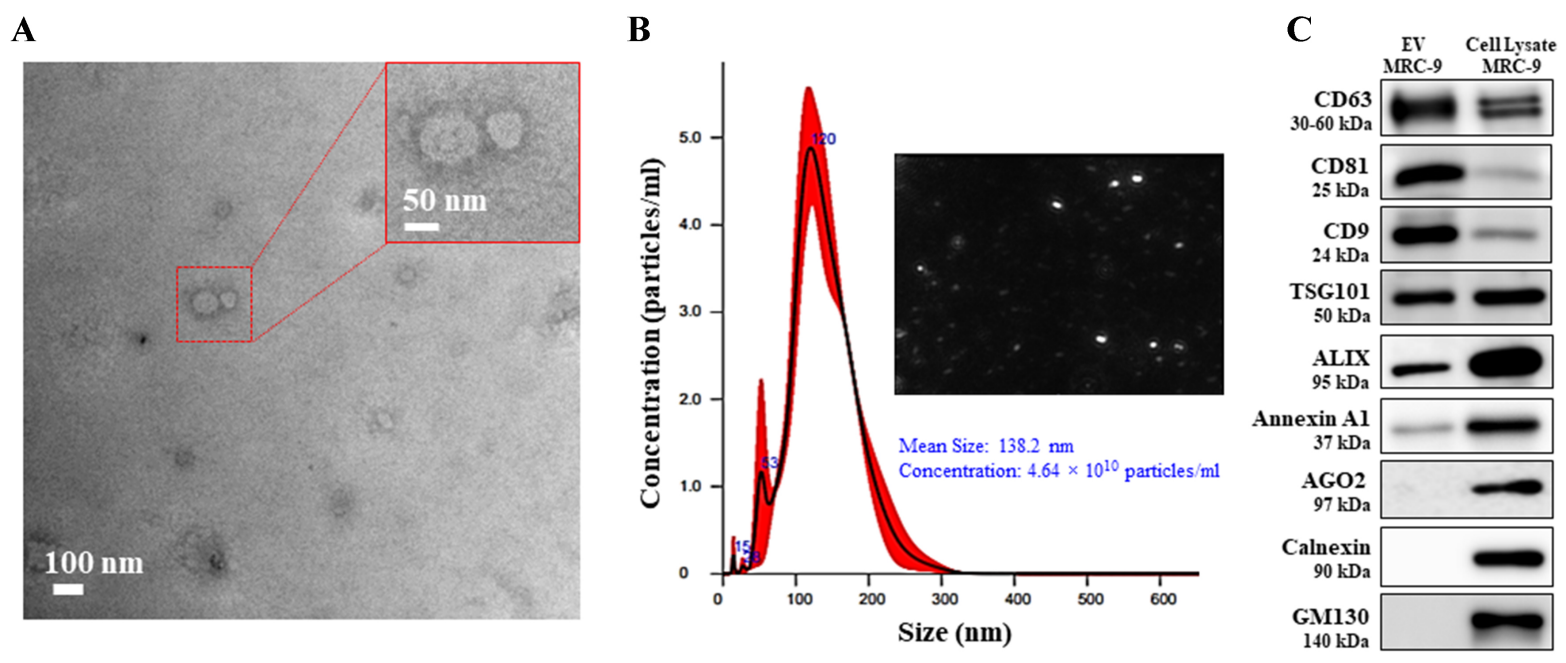
Characterization of EVs isolated from human lung fibroblast MRC-_9_ cell line. (A) TEM image (scale bar 100 nm) and the inset represents an enlarged image; (B) Histogram of NTA analysis and image of distributed EVs; (C) Western blot analysis for protein markers of EVs (CD63, CD81, CD9, TSG101, and Alix) and for markers discerning from microvesicles and non-EVs (Annexin A1, AGO2, calnexin and GM130) were compared with MRC-_9_ cell lysate that served as the positive reference. EVs: Extracellular vesicles; TEM: transmission electron microscopy; NTA: nanoparticle tracking analysis; TSG101: tumor susceptibility gene 101; Alix: ALG-2-interacting protein X; AGO2: argonaute 2; GM130: Golgi matrix protein 130.

The EVs isolated from MRC-_9_ cells were subjected to bio-physical characterization per criteria laid down by the International Society of Extracellular Vesicles (ISEV)^[38]^. Analysis of EVs by TEM and nanoparticle tracking analysis (NTA) showed particles were well dispersed without any aggregation and exhibited the characteristic double-layered cup-shape structure with a mean size of 138.2 nm [Figure 1A and B]. The smaller EV size observed in TEM (~100 nm; Figure 1A) compared with the size measured by NTA (138.2 nm; Figure 1B) is due to particle fixation during TEM analysis, which results in a reduced size. In contrast, NTA measures particles in Brownian motion and hence gives a relatively larger dynamic size. The number of EVs, as determined by NTA, was 4.64 × 10^10^ particles/mL, which was adequate for conducting the *in vitro* studies. Next, EV-specific markers recommended by minimal information for studies for extracellular vesicles (MISEV) were evaluated by western blot analysis^[38]^. As shown in Figure 1C, key EV markers such as CD63, CD81, CD9, TSG101, and Alix were detected in the EV preparation from MRC-_9_ cells. In contrast, Annexin A1, which is commonly associated with microvesicles^[39]^, was substantially less abundant in the EV sample compared to the MRC-_9_ cell lysate from which the EVs were isolated. This supports the notion that the purified EVs were largely free of microvesicle contamination. Furthermore, EV-negative markers, including AGO2, calnexin, and GM130, showed no detectable bands in the EV samples, confirming the purity of the EV preparation and being free from other contaminants^[38]^.

### Physicochemical properties show successful GNP-CDDP loading onto EVs to form EV-GNP-CDDP complex

**Figure 2:**
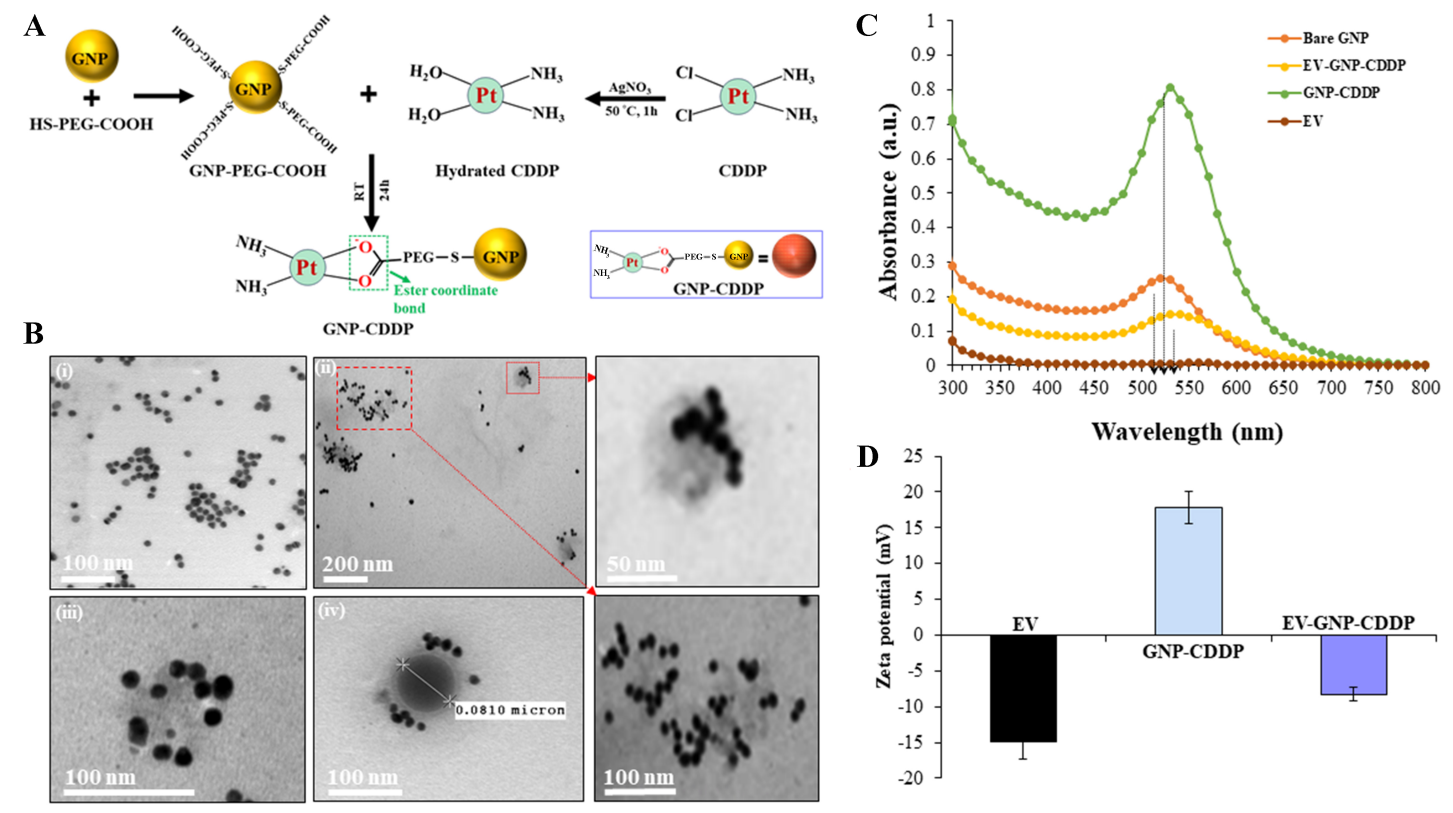
Synthesis and physicochemical characterizations of CDDP-functionalized GNPs and loading of CDDP-GNP into EVs. (A) Schematic illustration of the key stages of GNP-CDDP synthesis. This illustration was created using Microsoft PowerPoint; (B) TEM image of (i) GNPs and (ii-iv) EV-GNP-CDDP. The inset represents a boxed area showing an enlarged image; (C) Absorbance spectroscopy of EVs, bare GNPs, GNP-CDDP and EV-GNP-CDDP; (D) Zeta potential measurements at each step of preparing EV-GNP-CDDP. Bar graph represents the mean ± SD from three replicates (*n* = 3). CDDP: Cisplatin; GNPs: gold nanoparticles; EVs: extracellular vesicles; TEM: transmission electron microscopy; SD: standard deviation.

The GNP-CDDP synthesis detailing each major stage in the process is depicted in Figure 2A. GNPs of ~20 nm in size were synthesized and confirmed by TEM [Figure 2B] and absorption spectrum at 520 nm [Figure 2C] as previously described^[40]^. The GNPs were subsequently conjugated with hydrated CDDP via the pH-sensitive coordinate ester linkage (-COO-Pt) as described in the Supplementary Materials and shown in Figure 2A. The yield of hydrated CDDP conversion from CDDP was estimated to be ~74% and the CDDP conjugation efficiency to the GNPs was estimated to be ~33.12% *±* 3.04%, as determined by the OPDA assay. The CDDP-conjugated GNPs were labeled as GNP-CDDP.

Next, simple mixing of GNP-CDDP (1 mg equivalent of CDDP) with MRC-_9_-derived EVs (500 µg protein equivalent) with mild stirring at 37 °C for 2 h resulted in successful loading of GNP-CDDP onto EVs [Figure 2B and D]. TEM showed the double membrane structure of EVs and GNPs visible as dark dense spheres randomly distributed on the surface and lumen of the EVs. Surface charge analysis at pH 7.4 revealed that empty EVs had a negative charge (-14.97 mV) and GNP-CDDP possessed a positive surface charge (+17.78 mV; Figure 2D). In contrast, the surface charge of GNP-CDDP-loaded EVs showed a negative charge (-8.24 mV; Figure 2D). The observed intermediate negative surface charge of GNP-CDDP-loaded EVs is likely due to the electrostatic interaction between the negatively charged lipid membrane of EVs and the positively charged GNP-CDDP. This charge-based interaction likely contributes to loading GNP-CDDP into EVs. An alternative explanation for the GNP-CDDP loading onto EVs is that the EV lipid membrane is continuously fluid and that stirring at 37 °C disrupts and reorganizes the membrane, resulting in the uptake of GNP-CDDP by the EVs. The loading efficiency of GNP-CDDP on EVs was approximately 50% ± 17.5%, as determined by the OPDA assay. The observed drug loading variability of 17.5% likely arises from a combination of factors, including the high input concentration (50%), the intrinsic physicochemical properties of CDDP, and its interaction dynamics with the exosomal membrane. This concentration was selected to enhance loading efficiency while preserving EV integrity. Key parameters, such as incubation time, mixing method, and EV source, were carefully optimized to ensure consistent cargo incorporation. Overall, the achieved loading efficiency represents a calculated balance between maximizing drug payload and maintaining biological compatibility. Further, ICP-MS analysis demonstrated that the molar ratio of Gold/Platinum (Au/Pt) was 4.4 in the GNP-CDDP complex.

The conjugation of GNP-CDDP and loading on EVs was further confirmed with UV-Vis spectroscopy [Figure 2C] and FTIR spectroscopy [Supplementary Materials and Supplementary Figure 1]. The UV-Vis spectra measure the absorption maximum of GNPs and the absorption maximum shift when chemical conjugations or interactions occur^[41]^. As mentioned in Figure 2C, the EVs exhibited no absorbance, while bare GNPs showed an absorption maximum at 520 nm. In contrast, the addition of CDDP to GNP resulted in a shift of the absorption maximum of GNPs to 530 nm, indicating successful conjugation of CDDP to GNPs. The absorption maximum was further shifted to 540 nm when GNP-CDDP was loaded onto the EVs. The absorption peak of EV-GNP-CDDP complex confirmed successful conjugation of CDDP to GNP and their loading on EVs.

### Tumor targeting in tt-Mfn-EVs is conferred by insertion of Tf-DSPE lipid into EVs

**Figure 3:**
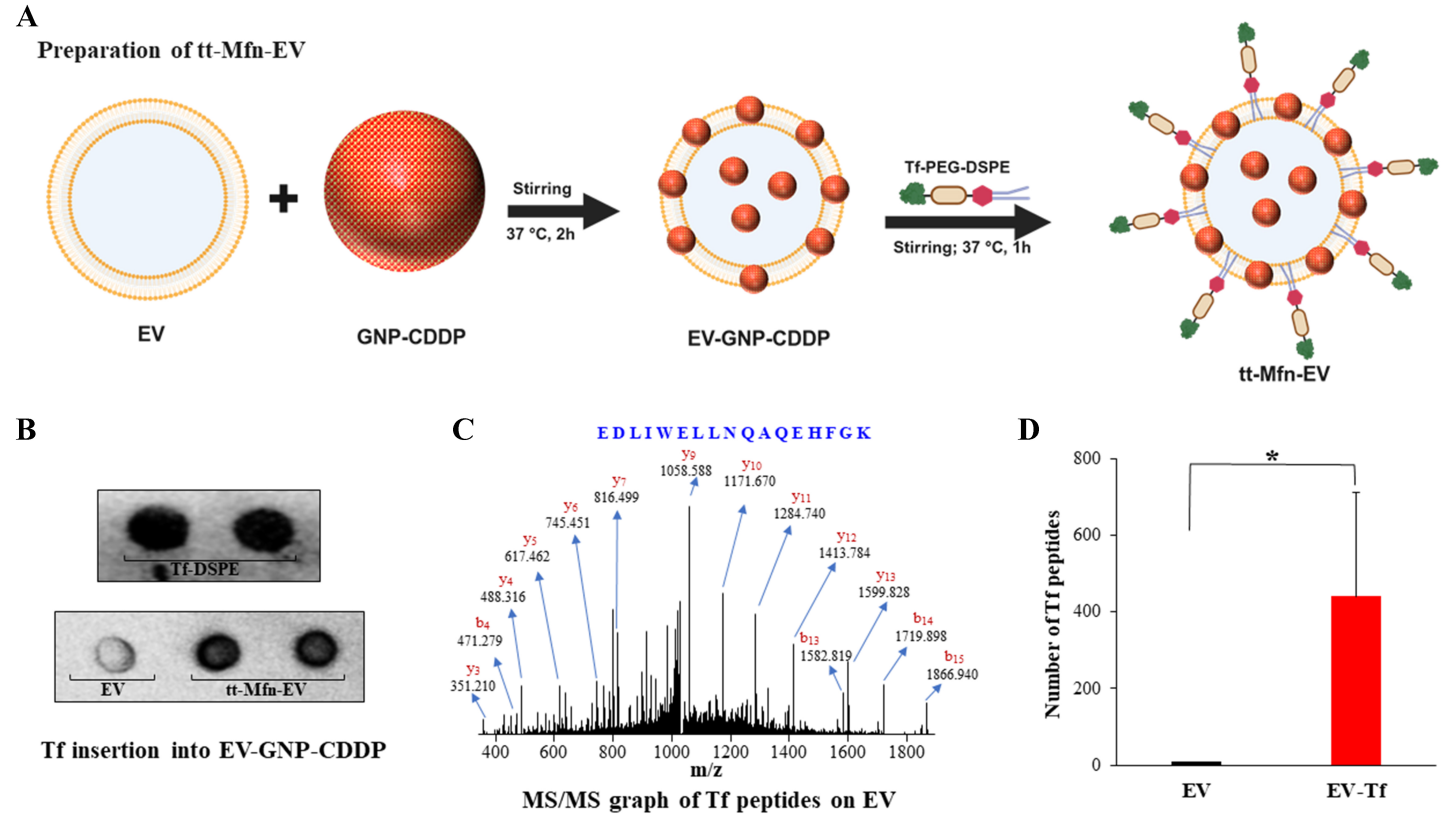
Preparation and validation of transferrin-conjugated EVs. (A) Schematic illustration of the preparation of functionalized tt-Mfn-EVs. This illustration was created using BioRender.com; (B) Dot blot images of activated transferrin conjugated DSPE lipid (Tf-DSPE), empty EVs, and Tf-DSPE-treated EVs loaded with GNP-CDDP (tt-Mfn-EVs); (C) MS/MS graph of transferrin peptide sequence in Tf-DSPE-treated EVs; (D) Quantification of transferrin peptide sequence on EVs and EV-Tf. The bar graph represents the mean ± SD from three replicates (*n* = 3). Statistical significance was assessed using an unpaired Student’s *t*-test, with *P* values indicated as ^*^P < 0.05. Created in BioRender. Ramesh, R. (2025) https://BioRender.com/yup6by2. EVs: Extracellular vesicles; tt-Mfn-EVs: tumor-targeted multifunctional extracellular vesicles; DSPE: 1,2-distearoyl-sn-glycero-3-phosphoethanolamine; GNP: gold nanoparticle; CDDP: cisplatin; MS: mass spectrometry; SD: standard deviation.

The process of functionalizing tt-Mfn-EVs is depicted in Figure 3A. Tumor-targeting ability of the multifunctional EVs towards TfR was generated by inserting Tf ligand using the lipid insertion method. A series of physical and chemical methods were used for generating tt-Mfn-EVs. First, hTf with 67-71 KDa MW was converted to thiolate transferrin (Tf-SH) through iminothiolane reagent (Traut’s reagent). This sulfhydryl modification enables chemical conjugation with the maleimide group present in the maleimide-PEG-DSPE lipid (2,941 MW). The Tf-SH is then chemically conjugated to the DSPE lipid through the maleimide group. The conjugation of Tf to DSPE lipid was confirmed with dot blot analysis [Figure 3B]. Next, Tf-DSPE lipid was incubated with EVs loaded with GNP-CDDP at 37 °C for 2 h with mild stirring, resulting in the insertion of Tf-lipid into the EV membrane. The rationale for utilizing this method of ligand insertion was based on the fact that the double-membrane structure of EVs is in a dynamic state, akin to liposomes when incubated at 37 °C^[42,43]^. The insertion of Tf-DSPE lipid into EVs was confirmed with a dot blot image and mass spectrometry. Dot blot analysis of tt-Mfn-EVs showed a dark contrast when compared to empty EVs to which Tf-DSPE was not added confirming Tf insertion into the EVs [Figure 3B]. Tf-DSPE lipid served as positive control in this assay. Further, the Tf sequence and molecular structure on EVs were confirmed with mass spectrometry [Figure 3C]. The mass spectrometry results confirmed the presence of the transferrin sequence in EV-Tf samples, matching the reported transferrin peptide sequence in the literature. Unique peptides from the transferrin protein were identified by both high-resolution mass spectrometry (MS) on parent peptide ions, and MS/MS on the fragments. The MS/MS spectrum identified EDLIWELLNQAQEHFGK as a unique peptide sequence belonging to transferrin. Addition of 4, 6 and 10 µg of transferrin resulted in the conjugation of 204, 383, and 736 transferrin peptide sequences, respectively, and an average of 441 transferrin peptide sequences conjugated to the EV-Tf group [Figure 3D], whereas free EVs contained eight transferrin peptide sequences. All the highly abundant peaks were confirmed as the fragments of transferrin peptides and the increase in the number of unique peptides identified by analyzing the baric tags in the MS spectrum suggested the successful conjugation of transferrin on EVs.

### Drug release from EV-GNP-CDDP is enhanced under *in vitro* acidic conditions

**Figure 4:**
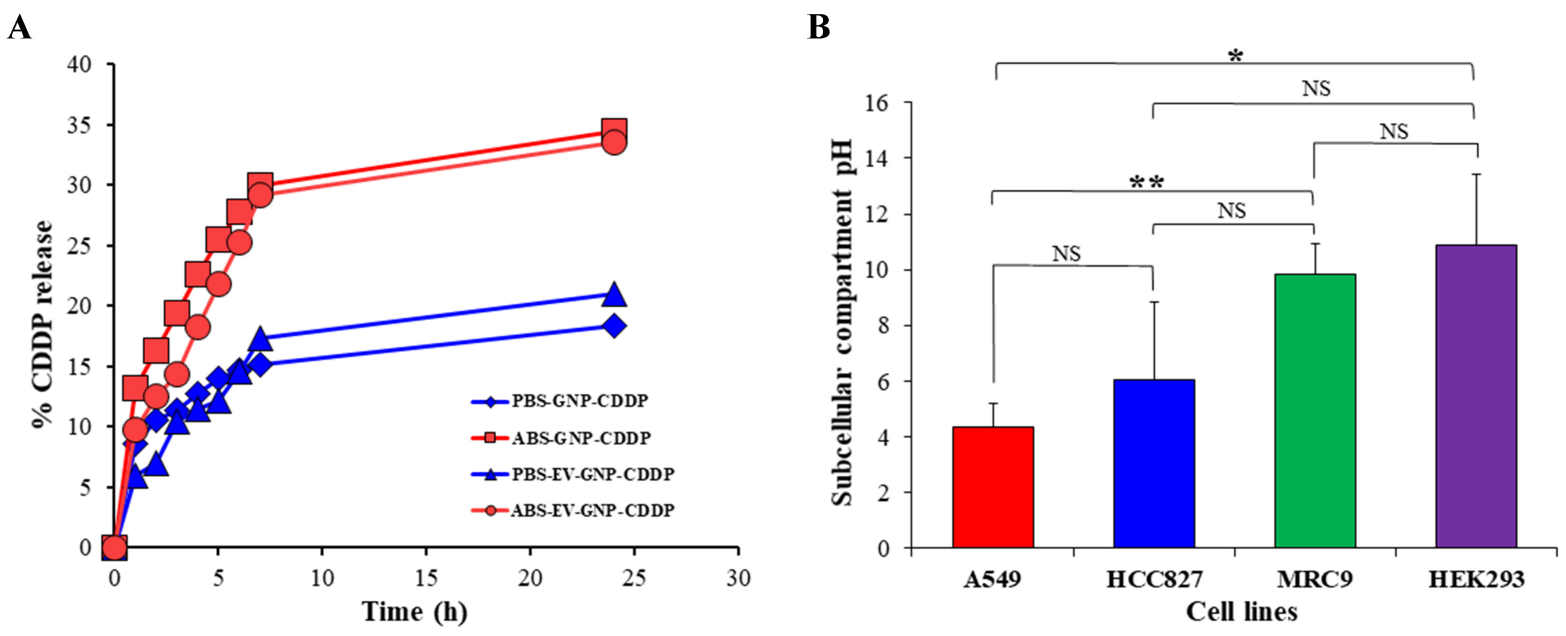
Assessment of *in vitro* drug release kinetics and intracellular pH. (A) CDDP release from GNP-CDDP and EV-GNP-CDDP was greater in acetate buffer (ABS, pH 5.5) compared to drug release in PBS buffer (PBS, pH 7.4); (B) Subcellular compartment pH measurement showed that tumor (A_549_, HCC827) cells were acidic compared to normal (MRC-_9_, HEK_293_) cells. The bar graph represents the mean ± SD from three replicates (*n* = 3). Statistical significance was assessed using an unpaired Student’s *t*-test, with *P* values indicated as ^*^P < 0.05, ^**^P < 0.01, NS denotes not significant. CDDP: Cisplatin; GNP: gold nanoparticle; EV: extracellular vesicle; ABS: acetate buffer; PBS: phosphate buffer; HEK_293_: human embryonic kidney 293; SD: standard deviation.

Since the objective of the study is to have selective and sustained drug release under acidic conditions, a common feature of the intracellular tumor milieu and the surrounding tumor microenvironment, CDDP was conjugated to GNPs through the metal coordination (ester-Pt) bond. This facilitates the ester linkage susceptible to cleavage under acidic conditions and release of CDDP from GNPs. To determine the selective drug release under acidic conditions, release kinetic studies were carried out in two different pH buffer conditions, ABS (pH 5.5) and PBS (pH 7.4), to mimic the tumor microenvironment and physiological environment, respectively. Incubating GNP-CDDP and EV-GNP-CDDP in ABS buffer solution (pH 5.5) resulted in about 34% and 33% of drug release, respectively, compared to drug release of 18% and 20% for GNP-CDDP and EV-GNP-CDDP, respectively, in PBS (pH 7.4; Figure 4A). The higher release of the drug in a lower pH environment offers the advantage of selective and specific drug release in the acidic tumor environment than in the surrounding normal physiological environment, thereby significantly reducing non-specific toxicity to normal tissues.

### Intracellular microenvironment of cancer cells is more acidic than that of non-tumor cells

The intracellular pH in tumor cells and in the surrounding tumor microenvironment is reported to be acidic in nature due to higher glycolysis and more glutathione concentration when compared to the normal physiological environment^[44-46]^. Based on these reports, pH-sensitive linkers have been used for selective drug release inside the tumor cells for achieving greater tumor cell killing with reduced cytotoxicity to normal cells^[47,48]^. Since the objective of this study was also to use selective drug release to tumor cells, we assessed the subcellular compartment pH of NSCLC (A_549_ and HCC827) cell lines, MRC-_9_ and normal human embryonic kidney (HEK_293_) cell lines. As shown in Figure 4B, the subcellular compartment pH of A_549_ and HCC827 was around 4.3 and 6.0, respectively. In contrast, the subcellular compartment pH of MRC-_9_ and HEK_293_ was 9.8 and 10.8, respectively. The fluorescence-based subcellular compartment pH measurements using pHrodo^TM^ Red AM revealed enhanced signal intensity in tumor cells, indicative of increased acidic vesicular burden, such as lysosomes and autolysosomes. This reflects compartmental acidification associated with elevated autophagic flux rather than whole-cell cytosolic pH^[49]^. While the reverse pH gradient is a hallmark of cancer metabolism^[50]^, our data underscore these localized acidic microenvironments. These results clearly indicate that cancer cells have an acidic subcellular compartment pH, and the use of pH-sensitive linker coordinate ester-Pt linkage in EV-GNP-CDDP complexes should be advantageous and facilitate selective drug release, resulting in enhanced tumor cell killing with minimal toxicity to normal cells.

### Uptake of tt-Mfn-EV by lung cancer cells

To assess the benefit of using tumor-targeted EVs, we conducted cell uptake studies in A_549_ lung cancer cells. A_549_ cells treated with GNP-CDDP, EV-GNP-CDDP and tt-Mfn-EVs were subjected to ICP-MS analysis at 24 h after treatment. ICP-MS showed intracellular concentrations of platinum metal in GNP-CDDP-, non-targeted EV-GNP-CDDP- and tt-Mfn-EV-treated cells were 1.5, 7.24, and 12.12 parts per million (PPM), respectively. Similarly, intracellular uptake of gold in GNP-CDDP-, non-targeted EV-GNP-CDDP- and tt-Mfn-EV-treated cells was 15.86, 83.79 and 100.14 PPM, respectively. These results showed that an increased uptake of GNP-CDDP occurred when delivered via EVs compared to non-EV delivery. Additionally, incorporating a tt-Mfn-EVs greatly increased the cellular uptake compared to non-targeted EVs (EV-GNP-CDDP).

Based on the ICP-MS analysis for GNPs and CDDP, we also calculated the number of Au atoms, Pt atoms and EVs present inside the treated cells using Avogadro’s number. The highest number of Pt atoms was observed in the tt-Mfn-EV treatment group (1.56381 × 10^20^ of Pt atoms), followed by non-targeted EV-GNP-CDDP (9.33242 × 10^19^ of Pt atoms) and GNP-CDDP (1.93216 × 10^19^ of Pt atoms). The number of Au atoms inside the cells also followed the trend similar to the Pt atoms and showed that the tt-Mfn-EV treatment group had the highest number of Au atoms (1.87635 × 10^21^), and EV-GNP-CDDP and GNP-CDDP showed 1.57008 × 10^21^ and 2.97209 × 10^20^ of Au atoms, respectively. For determining the number of EVs present inside the cells after 24 h of treatment, we calculated the number of EVs initially added to the cells, combined with the number of Pt and Au atoms determined by ICP-MS analysis. Based on this method, we determined that approximately 1.95 × 10^9^ EVs were present inside the tt-Mfn-EV-treated cells and were higher compared to non-targeted EV-GNP-CDDP-treated cells (1.16 × 10^9^ EVs).

**Figure 5:**
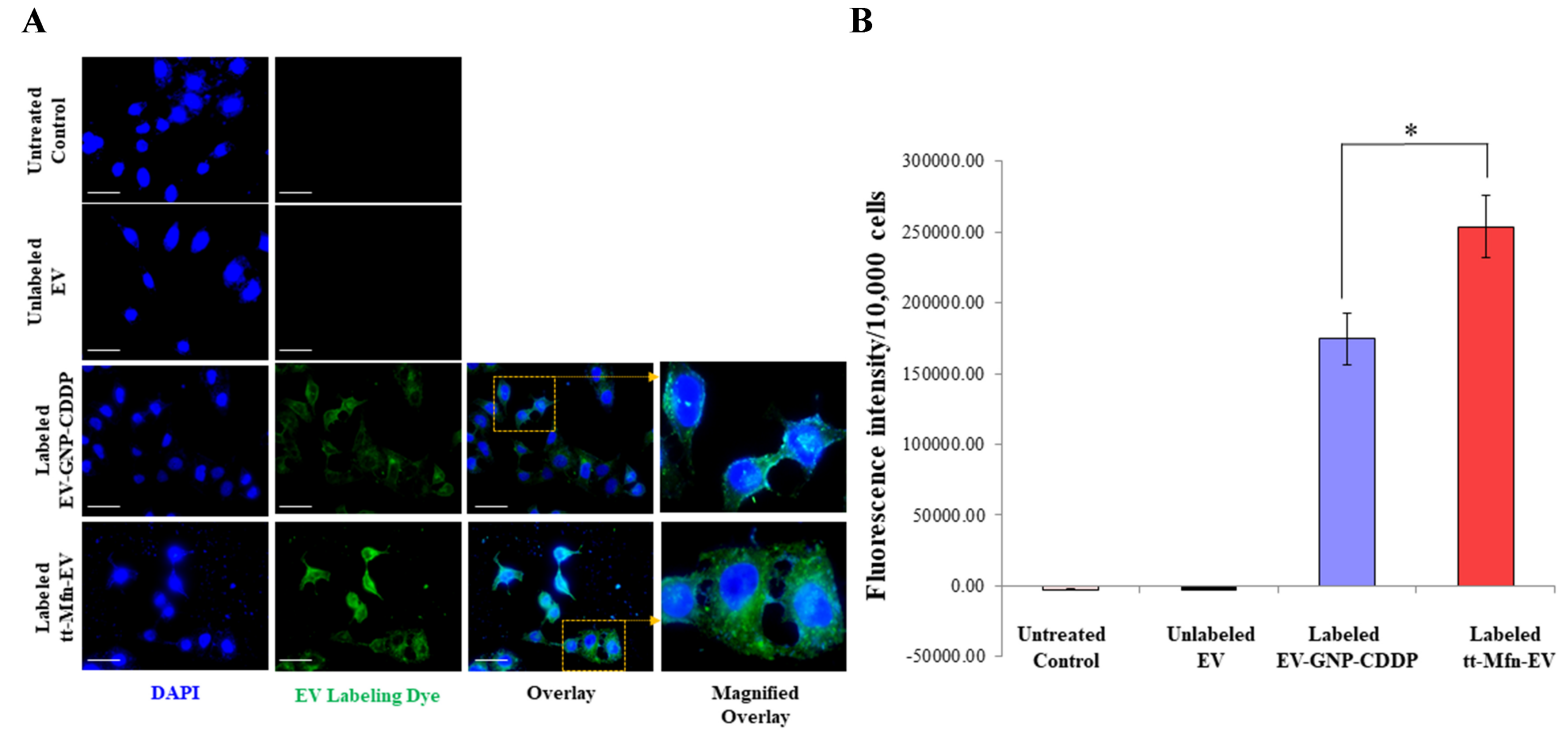
Measurement of tumor cell uptake of tt-Mfn-EVs. (A) Fluorescence microscopy images of A_549_ cells at 24 h after treatment with fluorescently labeled EV-GNP-CDDP and tt-Mfn-EVs. Untreated cells and cells treated with unlabeled EVs served as controls*.* Magnified overlay image showed a shift from punctate to diffuse cytoplasmic green fluorescence in A_549_ cells after 24 h, confirming efficient EV uptake. Fluorescein (green)-labeled EVs; DAPI (blue)-nucleus. Magnification, 60×; scale bar: 25 µm; (B) Quantification of cellular uptake based on fluorescence intensity in cells treated with labeled EV-GNP-CDDP and tt-Mfn-EVs, compared to unlabeled EVs and untreated controls. Fluorescent signal confirmed uptake in both labeled groups, with the highest uptake observed in tt-Mfn-EV-treated cells. The bar graph represents the mean ± SD from three replicates (*n* = 3). Statistical significance was assessed using an unpaired Student’s *t*-test, with *P* values indicated as ^*^P < 0.05. tt-Mfn-EVs: Tumor-targeted multifunctional extracellular vesicles; GNP: gold nanoparticle; CDDP: cisplatin; DAPI: 4, 6-diamidino-2-phenylindole; SD: standard deviation.

To further confirm the cell uptake of EVs, fluorescence microscopy was performed in the A_549_ lung cancer cell line using fluorescently labeled EVs. In this experiment, EV-GNP*-*CDDP and tt-Mfn-EVs were fluorescently labeled with ExoGlow^TM^-Membrane EV Labeling dye and added to A_549_ cells (7.5 × 10^10^ particles equivalent to 100 μg protein). Cells treated with unlabeled EVs and cells receiving no treatment served as controls. As shown in Figure 5A, the addition of fluorescently labeled EV-GNP-CDDP and tt-Mfn-EVs showed punctate to diffuse cytoplasmic intracellular green fluorescence in A_549_ cells after 24 h of treatment. The tt-Mfn-EV group, however, showed the greatest intracellular fluorescence intensity when compared to the non-targeted EV-GNP-CDDP, unlabeled EVs and untreated control cells (Figure 5B; *P* < 0.05). Cells receiving no treatment and cells treated with unlabeled EVs showed no green fluorescence. These results demonstrated that EVs were efficiently taken up by tumor cells, and the addition of a tumor-targeted ligand increased the cell uptake. Also, as shown in Supplementary Figure 2, A_549_ cells treated with EV labeled using the ExoGlow^TM^-Protein EV Labeling Kit exhibited a distinct red fluorescence signal, while dye-only control and cells receiving no treatment showed no detectable fluorescence. These results confirm that the observed intracellular fluorescence originates specifically from EVs uptake rather than non-specific or artefactual staining. Collectively, the data support the robustness of the labeling strategy and demonstrate that transferrin functionalization enhances cellular internalization and amplifies therapeutic efficacy.

Next, we investigated EV uptake by normal cells. For this purpose, human normal MRC-_9_ fibroblast cells were treated with fluorescently labeled EV-GNP-CDDP and tt-Mfn-EVs and were compared to cells receiving no treatment. A significant increase in EV uptake was observed in EV-GNP-CDDP-treated cells compared to tt-Mfn-EV-treated cells (Supplementary Figure 3; *P* < 0.01). These results indicated that the use of tumor-targeted EVs reduced their uptake by normal cells, thus potentially reducing non-specific toxicity.

Finally, the use of EVs over conventional liposomes as drug delivery vehicles was assessed. Both EVs and liposomes conjugated to Tf and fluorescently labeled were added to A_549_ cells. Cells treated with unlabeled EVs were used as a control. As shown in Supplementary Figure 4A and B, Tf conjugated EV uptake by tumor cells was 3.7 times greater compared to Tf conjugated liposomes at 12 h after treatment (*P* < 0.01). The magnified overlay in Supplementary Figure 4A clearly demonstrates a transition from punctate to diffuse green fluorescence within the cytoplasm of A_549_ cells after 24 h of treatment, indicating efficient EV uptake by the tumor cells. Our results showed that tumor-targeted EVs are taken more efficiently than liposomes and thus could serve as an effective drug delivery vehicle for cancer treatment.

### tt-Mfn-EVs exert greater antitumor activity on lung cancer cell lines

The therapeutic efficacy studies of tt-Mfn-EVs were carried out in A_549_ and HCC827 lung cancer cell lines and compared to normal MRC-_9_ and HEK_293_ cell lines. Prior to start of the therapeutics studies, we first analyzed TfR expression by western blotting in three lung cancer cell lines (A_549_, HCC827 and H1299) and in two normal cell lines (MRC9 and HEK_293_). TfR expression was high in lung cancer cell lines compared to normal cell lines albeit the expression levels varied among the three cancer cell lines [Supplementary Figure 5A] and concurred with our previous findings^[32,37]^. Based on the TfR expression levels observed in the A_549_ and HCC827 cells used in the present study, we referred to these two cell lines as high and low TfR expressors, respectively^[32]^. H1229 cell lines that were not tested in the present study were referred to as medium TfR expressors. The high TfR expression observed among lung cancer cells supported TfR-targeted delivery strategies for cancer therapy^[32,37]^.

**Figure 6:**
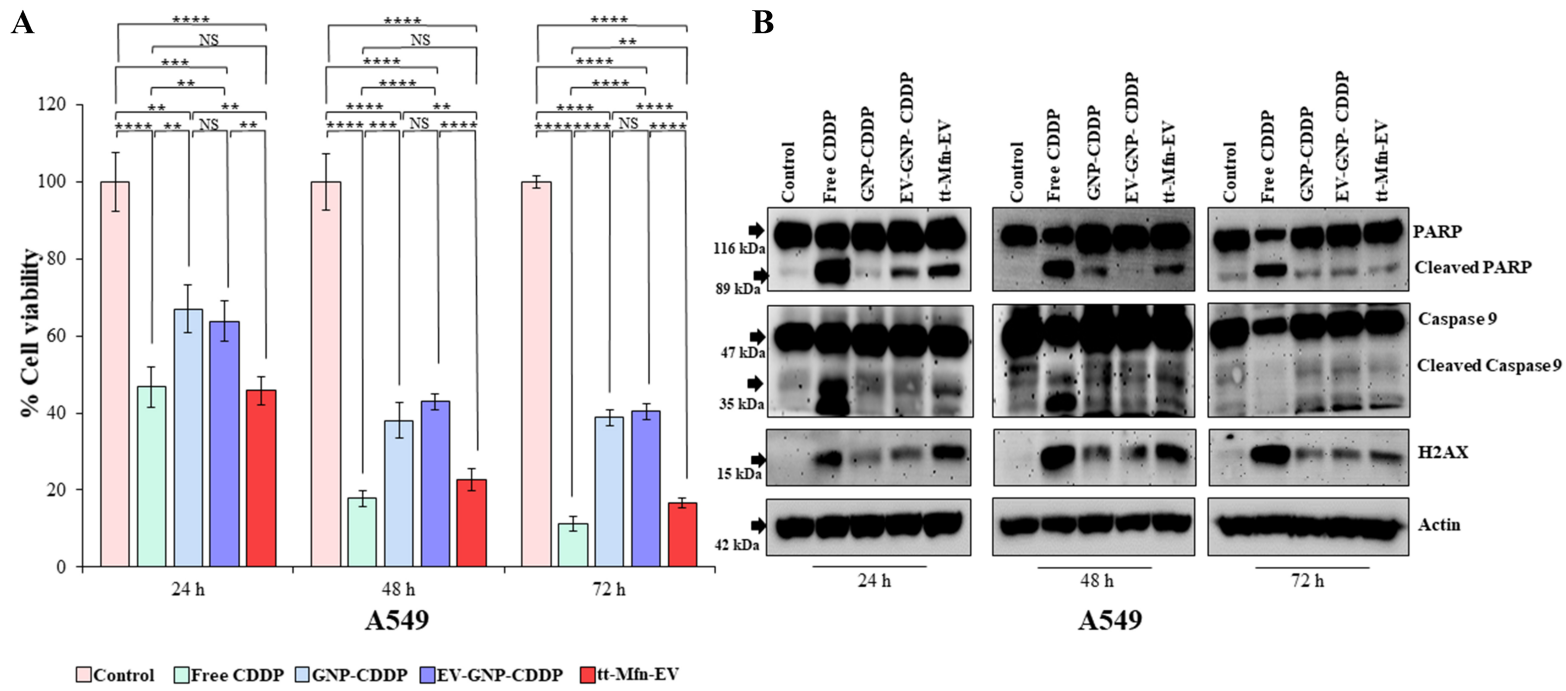
Therapeutic efficacy of tt-Mfn-EVs in A_549_ lung cancer cells. Cells were treated with tt-Mfn-EV, free CDDP, GNP-CDDP, EV-GNP-CDDP and assessed for cell viability and apoptosis markers at 24, 48 and 72 h after treatment. Cells receiving no treatment served as the control. (A) Cell viability showed that tt-Mfn-EV significantly and greatly reduced cell viability compared to GNP-CDDP, EV-GNP-CDDP treatments. Free CDDP, however, showed the highest cytotoxicity among all treatment groups; (B) Western blot analysis showed activation of apoptosis was highest in free CDDP treatment, followed by tt-Mfn-EV, EV-GNP-CDDP and GNP-CDDP. Bar graph represents the mean ± SD from three replicates (*n* = 3). Statistical significance was assessed using an unpaired Student’s *t*-test, with *P* values indicated as ^**^P < 0.01, ^***^P < 0.001, ^****^P < 0.0001, NS denotes not significant. tt-Mfn-EVs: Tumor-targeted multifunctional extracellular vesicles; CDDP: cisplatin; GNP: gold nanoparticle; SD: standard deviation.

Cells (A_549_ and HCC827) were treated with tt-Mfn-EVs and compared to EV-GNP-CDDP and GNP-CDDP. Additionally, free CDDP was included in the study as a positive control and for comparison with tt-Mfn-EVs. Cells receiving no treatment served as controls. Cells were harvested at various time points and analyzed for reduction in cell number across various treatments and for molecular markers of apoptotic cell death. As shown in Figure 6A, tt-Mfn-EV treatment showed the highest reduction in cell viability compared to EV-GNP-CDDP and GNP-CDDP at all time points tested. Additionally, the tt-Mfn-EV-mediated cytotoxicity progressively reduced the cell viability from 45.7% at 24 h to 22.7% at 48 h and 16.7% at 72 h. The addition of tumor-targeted ligand significantly increased the tumor cell killing anywhere from 18% at 24 h to 24% at 72 h compared to EV-GNP-CDDP (*P* < 0.0001). It is to be noted that EV-GNP-CDDP and GNP-CDDP treatment did exert significant cytotoxicity compared to untreated control cells (*P* < 0.0001) at the three time-points tested. Furthermore, the tt-Mfn-EV-mediated cytotoxicity (45.7% viable cells) was comparable to free CDDP (46.8% viable cells) at 24 h, albeit a slight increase in cell killing was observed with free CDDP at 48 h (17.7% viable cells) and 72 h (11.2% viable cells) compared to tt-Mfn-EVs.

Molecular studies investigating tt-Mfn-EV-mediated mechanism of cell death revealed that cells underwent apoptotic cell death as evidenced by the cleavage of PARP and Caspase-9 [Figure 6B]. The tt-Mfn-EV showed significant and greater activation of PARP and Caspase-9 compared with EV-GNP-CDDP and GNP-CDDP (*P* < 0.05; Figure 6B and Supplementary Figure 5B-D) at 24 and 48 h after treatment. At 72 h, a slight increase in PARP but not in Caspase-9 cleavage was observed in the tt-Mfn-EV treatment group compared to EV-GNP-CDDP and GNP-CDDP. The greatest activation of PARP and Caspase-9, however, was observed in the free CDDP-treated group at all time points tested except for cleaved Caspase-9 at 72 h. One plausible explanation for the lack of an increase in cleaved Caspase-9 in the tt-Mfn-EV treatment at 72 h is likely that most of the cells were already dead, similar to the CDDP treatment. This is supported by our inability to detect cleaved Caspase-9 in CDDP-treated cells as well.

Apart from apoptotic markers, we also analyzed Histone 2A variant X (H_2_AX), which is a DNA damage marker and frequently increased when treated with DNA-damaging agents such as CDDP. A significant increase in H_2_AX protein expression was observed in all treatment groups when compared to the untreated control (Figure 6B, Supplementary Figure 5B-D; *P* < 0.05). As anticipated, CDDP treatment induced the greatest DNA damage as evidenced by the highest increase in H_2_AX expression. The tt-Mfn-EV treatment, including CDDP, showed higher levels of H_2_AX expression compared to EV-GNP-CDDP and GNP-CDDP at 24 and 48 h. Increase of H_2_AX expression in tt-Mfn-EV, however, was significant compared to GNP-CDDP but not compared to EV-GNP-CDDP. These results demonstrate that tt-Mfn-EVs enhanced tumor cell killing compared to non-targeted EV-GNP-CDDP and GNP-CDDP and tumor-targeted EV-based drug delivery is beneficial for lung cancer treatment.

**Figure 7:**
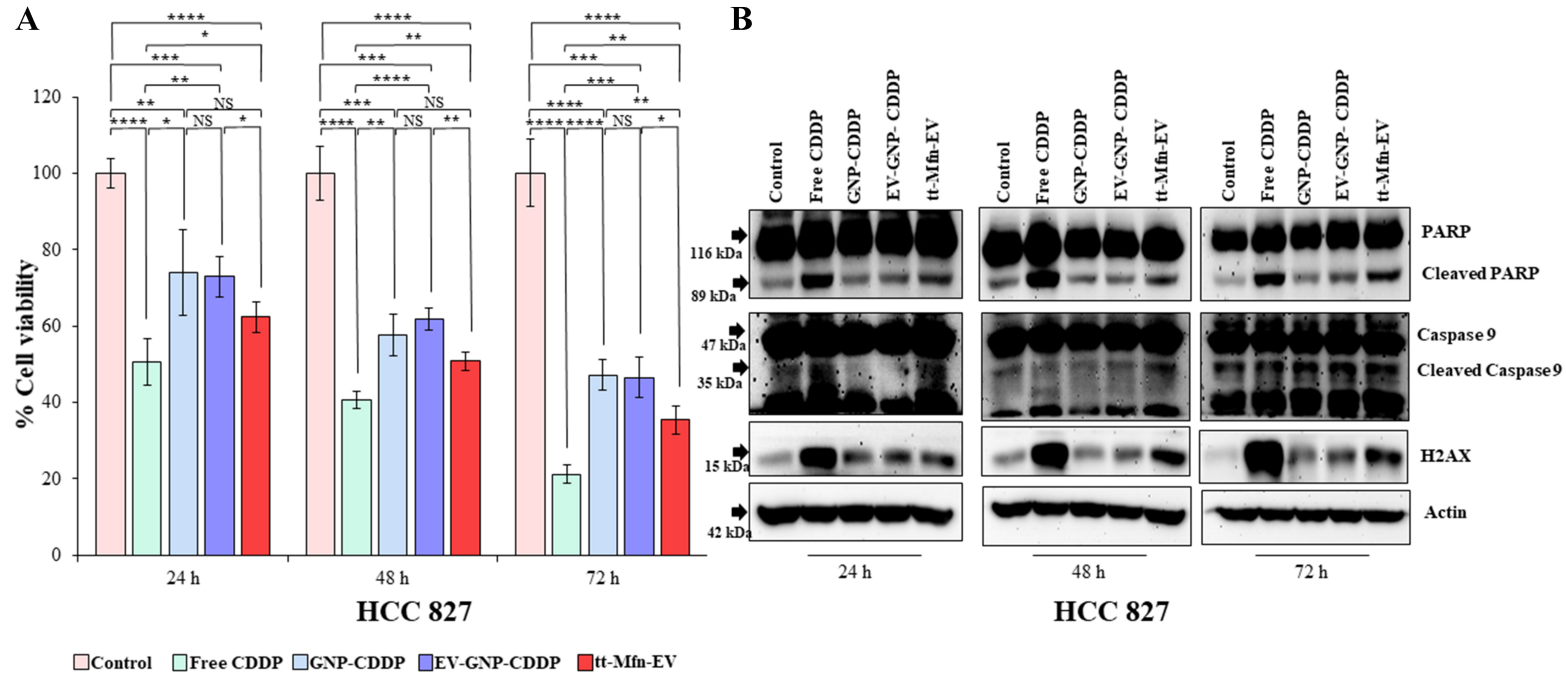
Therapeutic efficacy of tt-Mfn-EV in HCC827 lung cancer cells. Cells were treated with tt-Mfn-EV, free CDDP, GNP-CDDP, EV-GNP-CDDP and assessed for cell viability and apoptosis markers at 24, 48 and 72 h after treatment. Cells receiving no treatment served as the control. (A) Cell viability showed that tt-Mfn-EVs greatly reduced cell viability compared to GNP-CDDP, EV-GNP-CDDP treatments albeit significance was observed only at 48 h. Free CDDP however showed the highest cytotoxicity among all treatment groups; (B) Western blot analysis showed activation of apoptosis was highest in free CDDP treatment followed by tt-Mfn-EV, EV-GNP-CDDP and GNP-CDDP. The bar graph represents the mean ± SD from three replicates (*n* = 3). Statistical significance was assessed using an unpaired Student’s *t*-test, with *P* values indicated as ^*^P < 0.05, ^**^P < 0.01, ^***^P < 0.001, ^****^P < 0.0001, NS denotes not significant. tt-Mfn-EV: Tumor-targeted multifunctional extracellular vesicle; CDDP: cisplatin; GNP: gold nanoparticle; SD: standard deviation.

Next, the efficacy of tt-Mfn-EVs was tested in HCC827 lung cancer cells that express relatively low levels of TfR compared to A_549_ cells. All treatment groups (free CDDP, EV-GNP-CDDP, GNP-CDDP and tt-Mfn- EVs) showed significant inhibitory activity to cell viability compared to the untreated control group (Figure 7A; *P* < 0.05). Free CDDP treatment, however, showed the greatest inhibitory activity at all time points tested akin to that observed in A_549_ cells. In HCC827 cells, tt-Mfn-EV treatment showed greater inhibitory activity compared to EV-GNP-CDDP and GNP-CDDP groups. However, unlike in A_549_ cells, tt-Mfn-EV treatment showed no significant inhibitory activity compared to EV-GNP-CDDP and GNP-CDDP groups. Although significant inhibitory activity was observed between tt-Mfn-EV and EV-GNP-CDDP treatments at 48 h (*P* < 0.01), no significant inhibitory activity was observed when GNP-CDDP was compared with EV-GNP-CDDP and tt-Mfn-EV treatments. The overall difference in cell viability reduction between tt-Mfn-EV versus EV-GNP-CDDP or GNP-CDDP groups ranged between 11%-12% for any given time point and was significant.

Analysis for activation of apoptotic and DNA damage response markers showed increased cleavage of PARP and Caspase-9 and increased H_2_AX expression in all the treatment groups compared to the untreated control group [Figure 7B and Supplementary Figure 6]. However, rank ordering from the highest to lowest activation and expression for each of these proteins was in the following order: CDDP > tt-Mfn-EV > EV-GNP-CDDP > GNP-CDDP.

To further validate that tt-Mfn-EV-mediated inhibitory activity on cell viability results in apoptosis, an Annexin V/PI assay was conducted in both A_549_ and HCC827 cell lines and analyzed at 24 h after treatment. In A_549_ cells, tt-Mfn-EV treatment resulted in 61.6% of apoptotic cells (Supplementary Figure 7; *P* < 0.0001) compared to untreated control cells. Non-targeted EV-GNP-CDDP and GNP-CDDP treatment produced 5.35% and 8.65% of apoptosis, respectively, which was not significant compared to untreated cells. Free CDDP treatment produced the greatest Annexin V positive cells (65.1%; *P* < 0.0001) compared to untreated control cells. A similar trend in Annexin V-positive cells was observed in HCC827 cells, albeit to a lesser extent than that observed in A_549_ cells. The tt-Mfn-EV treatment resulted in 21.3% of cells undergoing apoptosis, while EV-GNP-CDDP and GNP-CDDP treatments resulted in 9.5% and 11.3% of apoptotic cells, respectively.

The greater cytotoxicity exerted by tt-Mfn-EV in A_549_ cells compared with HCC827 cells can be attributed to two factors: (1) the higher expression of TfR in A_549_, which leads to increased tt-Mfn-EV uptake and consequently higher intracellular CDDP levels that promote DNA damage and cell death; and (2) the more acidic intracellular pH in A_549_ cells (pH 4.3) compared with HCC827 cells (pH 6.0), which results in faster cleavage of the Pt-ester bond and more rapid CDDP activation, thereby enhancing tumor cell killing. Thus, exploiting the differences in pH and receptor expression contributed to the higher therapeutic efficacy in A_549_ cells than in HCC827 cells. Collectively, our results showed that transferrin-targeted EV drug delivery was more effective in inducing cytotoxicity and apoptotic cell death than the non-targeted treatment groups, which concurred with the cell viability and western blotting results presented in this study.

### TfR blocking shows that tt-Mfn-EV-mediated cytotoxicity is specific and receptor-mediated

**Figure 8:**
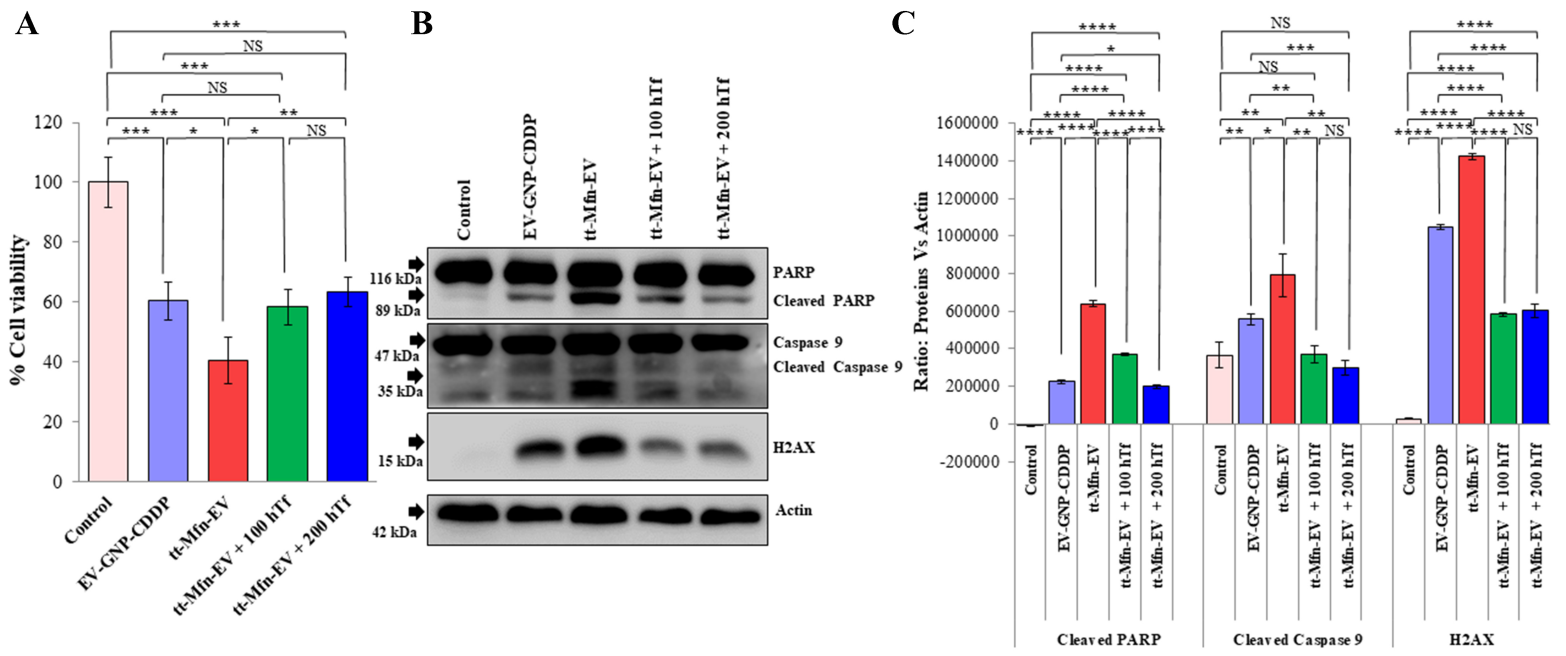
tt-Mfn-EV cell uptake is receptor-mediated and specific. TfR overexpressing A_549_ lung tumor cells were treated with tt-Mfn-EV in the presence or absence of human transferrin (Tf) and assessed for (A) Cell viability and (B) Molecular markers of apoptosis by Western blotting at 24 h after treatment. The hTf was added in two different concentrations (100 and 200 µg) 1 h prior to tt-Mfn-EV treatment. EV-GNP-CDDP-treated cells and untreated cells served as controls; (C) Quantification graphs of corresponding western blot analysis of cleaved PARP, cleaved caspase 9 and H_2_AX normalized against actin. Bar graphs represent the mean ± SD from three replicates (*n* = 3). Statistical significance was assessed using an unpaired Student’s *t*-test, with *P* values indicated as ^*^P < 0.05, ^**^P < 0.01, ^***^P < 0.001, ^****^P < 0.0001, NS denotes not significant. tt-Mfn-EV: Tumor-targeted multifunctional extracellular vesicle; TfR: transferrin receptor; hTf: human transferrin; GNP: gold nanoparticle; CDDP: cisplatin; PARP: poly (ADP-ribose) polymerase; H_2_AX: Histone 2A variant X; SD: standard deviation.

While cell viability and molecular studies showed that tt-Mfn-EV-mediated drug delivery was beneficial and exerted greater antitumor activity compared to non-targeted EVs, receptor specificity and selectivity remained to be elucidated. For this purpose, we conducted receptor-blocking studies in the presence of an excess of exogenous hTf. A_549_ tumor cells were incubated with hTf at two different concentrations (100 and 200 µg/well) for 1 h prior to the addition of tt-Mfn-EV. Cells treated with EV-GNP-CDDP only and untreated cells served as controls. At 24 h after treatment, the cells were collected and subjected to cell viability and molecular analysis by western blotting. As observed, tt-Mfn-EV treatment resulted in a significant reduction in cell viability (40% viable cells) compared to EV-GNP-CDDP treatment (60.3% viable cells) and untreated control cells (Figure 8A, *P* < 0.001). However, the tumor cell cytotoxicity of tt-Mfn-EV was significantly abrogated in the presence of hTf (Figure 8A, *P* < 0.05). A dose-dependent mitigation of tt-Mfn-EV cytotoxicity was observed in the presence of 100 µg (58.4% viable cells) and 200 µg (63.3% viable cells) of hTf. These results indicated that tumor cell uptake of tt-Mfn-EVs specifically occurred through the TfR, resulting in the observed antitumor activity.

Corroborating our cell viability results, the western blot data showed that tt-Mfn-EV-mediated induction of apoptosis was significantly reduced in the presence of exogenous hTf [Figure 8B and C]. Activation of both Caspase-9 and PARP, as measured by their protein cleavage, was significantly reduced in the presence of hTf, with greater reduction observed with 200 μg hTf (*P* < 0.001). Additionally, H_2_AX protein expression was also significantly reduced in the presence of hTf. However, the differences in H_2_AX expression in the presence of two hTf concentrations were not significant [Figure 8B and C]. The study results demonstrate that tt-Mfn-EV uptake by tumor cells is receptor-mediated and occurs specifically via the TfR and blocking TfR with exogenous Tf ligand abrogates the cell cytotoxicity. Thus, tumor-targeted drug delivery using EVs offers the benefit of selectively targeting TfR-overexpressing tumor cells to produce efficient antitumor activity.

### tt-Mfn-EV shows reduced toxicity towards normal lung and kidney cell lines

Our goal was to develop an EV-based delivery system that selectively targets lung cancer cells while minimizing toxicity to normal tissues. To assess this, we evaluated the cytotoxicity of tt-Mfn-EV in MRC-_9_ and HEK_293_ cell lines. Specifically, these normal cell lines were used to assess the cytotoxicity of the tt-Mfn-EV nanomaterials. This approach allowed us to evaluate not only lung-targeted cytotoxic effects but also potential nephrotoxicity, a well-documented side effect of CDDP treatment. These normal cells also enabled a comparative analysis of TfR sensitivity, which is significantly lower than in cancer cells, offering insight into the selectivity of our targeted delivery system.

**Figure 9:**
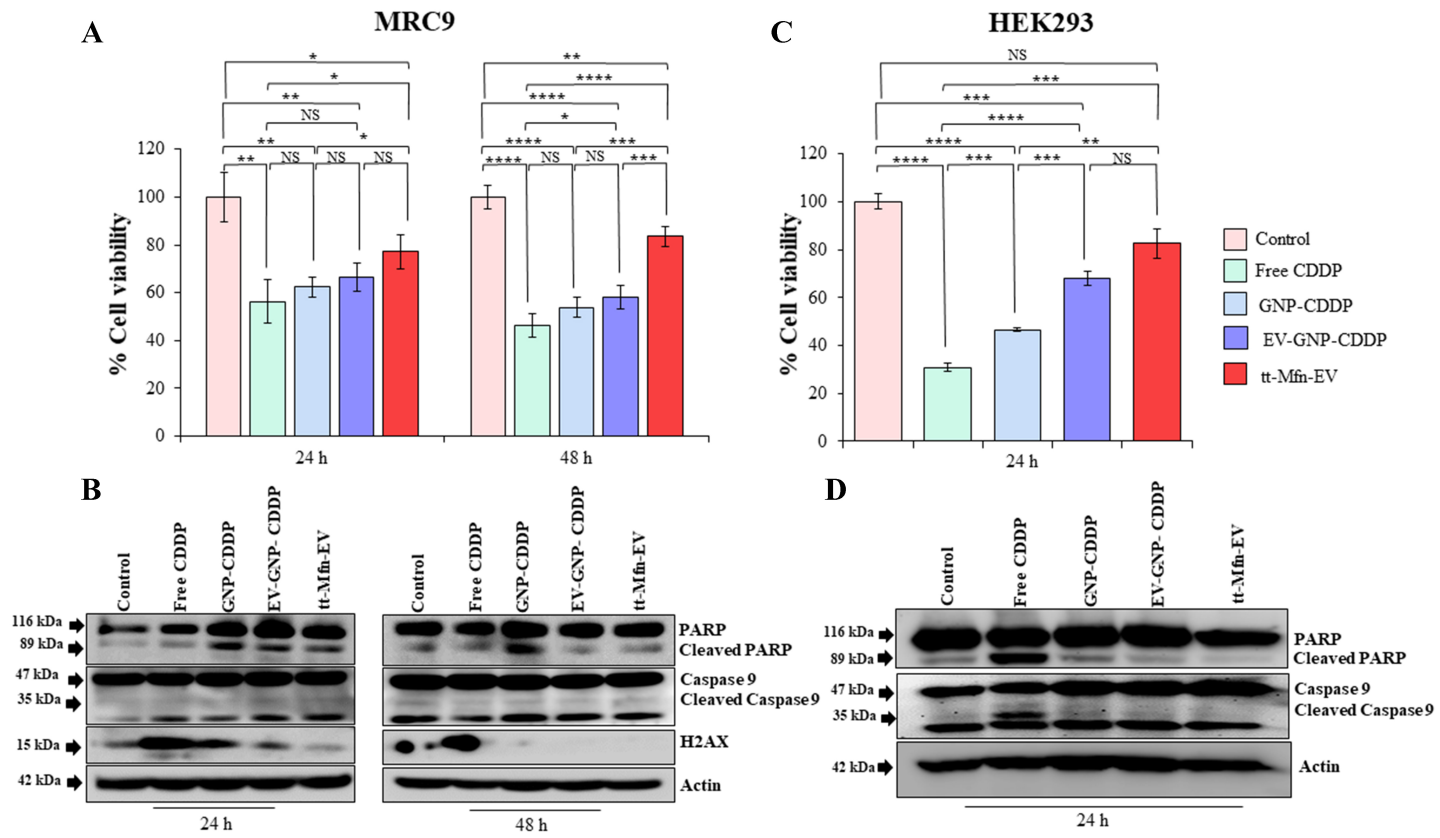
tt-Mfn-EV cytotoxicity assessment in normal cells. (A and B) Human normal lung fibroblast MRC-_9_ cells, and (C and D) human embryonic kidney (HEK)293 cells were treated with free CDDP, GNP-CDDP, EV-GNP-CDDP and tt-Mfn-EV and analyzed for cell viability and molecular markers by western blotting. The effect of tt-Mfn-EV treatment on MRC-_9_ was determined at 24 and 48 h, and on HEK_293_ at 24 h after treatment. Bar graphs represent the mean ± SD from three replicates (*n* = 3). Statistical significance was assessed using an unpaired Student’s *t*-test, with *P* values indicated as ^*^P < 0.05, ^**^P < 0.01, ^***^P < 0.001, ^****^P < 0.0001, NS denotes not significant. tt-Mfn-EV: Tumor-targeted multifunctional extracellular vesicle; HEK_293_: human embryonic kidney 293; CDDP: cisplatin; GNP: gold nanoparticle; SD: standard deviation.

In MRC-_9_ cells, treatment with free CDDP produced the greatest and significant cytotoxicity at both 24 h (56.2% viable cells) and 48 h (46.2% viable cells) followed by GNP-CDDP (62.5% and 53.73% viable cells) and EV-GNP-CDDP (66.6% and 58.2% viable cells) compared to untreated control cells (Figure 9A; *P* < 0.05). In contrast, tt-Mfn-EV treatment showed 77% and 83.5% of viable cells at 24 and 48 h, respectively, compared to untreated control cells. The cell viability results demonstrated that tt-Mfn-EV significantly reduced cytotoxicity compared to all other treatment groups, in particular, free CDDP, and a feature preferred in cancer therapy. Correlating with the cell viability results, the western blot data showed that tt-Mfn-EV treatment markedly reduced PARP and Caspase-9 cleavage, along with a reduction in the expression of the DNA damage marker H_2_AX [Figure 9B and Supplementary Figure 8]. Although the expression of cleaved PARP, Caspase-9, and H_2_AX varied among the treatment groups, it was nevertheless markedly higher than in the untreated control [Figure 9B and Supplementary Figure 8].

Next, we examined the toxicity of tt-Mfn-EVs on normal HEK_293_ kidney cells. The rationale is that nephrotoxicity is one of the major side effects of CDDP treatment^[51]^. As shown in Figure 9C, tt-Mfn-EV treatment resulted in 82.5% viable cells (17.5% cell toxicity) compared to untreated control cells. In contrast, treatment with free CDDP, GNP-CDDP and EV-GNP-CDDP resulted in 30.8%, 46.5% and 68% viable cells compared to untreated control cells at 24 h after treatment [Figure 9C]. Of interest to note is that tt-Mfn-EV significantly mitigated free CDDP-mediated toxicity by 51.7% (*P* < 0.001). Our data show that incorporating CDDP into GNP via a pH-selective linker results in reduced CDDP cytotoxicity, which is further improved by encapsulating GNP-CDDP into EVs. Thus, selective drug release under acidic conditions commonly observed in the tumor microenvironment using tt-Mfn-EVs should be efficacious and concurrently safe by reducing non-specific toxicity to normal cells.

Finally, western blotting analysis concurred with the cell viability data and showed a significant reduction in the cleavage of PARP and Caspase-9 upon tt-Mfn-EV treatment compared to free CDDP [Figure 9D and Supplementary Figure 9]. While GNP-CDDP treatment caused a significant reduction in PARP compared with free CDDP, levels remained relatively higher than in EV-GNP-CDDP and tt-Mfn-EV treatments (*P* < 0.0001), indicating that loading GNP-CDDP into EVs mitigates CDDP-induced toxicity. Cleavage of Caspase-9 was also reduced in GNP-CDDP and EV-GNP-CDDP treatments compared to CDDP treatment. The reduced toxicity observed with tt-Mfn-EV in normal MRC-_9_ and HEK_293_ cells is likely due to the low TfR expression levels compared to TfR expression in tumor cells, which curtails receptor-mediated endocytosis^[32,52]^. Additionally, the high alkaline intracellular pH observed in MRC-_9_ and HEK_293_ cells prevents hydrolysis of the pH-sensitive ester linker to release CDDP from GNP, thereby reducing CDDP-mediated toxicity. In conclusion, our data demonstrate that EV-based drug delivery offers benefits in reducing non-specific toxicity towards normal cells and incorporating a pH-selective linker for CDDP release from GNP enables selective drug release under acidic conditions, a feature commonly observed in tumor cells and tumor microenvironment but not in surrounding normal tissue.

## DISCUSSION

In the present study using a combination of nanotechnology and EV technology, we produced and tested tt-Mfn-EV-based drug delivery systems for lung cancer treatment. Our study results showed that exploiting receptor-mediated tumor cell targeting along with differences in intracellular pH between tumor cells and normal cells was advantageous in achieving selective and enhanced tumor cell killing while sparing normal cells. Additionally, CDDP-induced nephrotoxicity was also circumvented, which otherwise limits the drug’s use in the clinic for cancer treatment. Another potential advantage of tt-Mfn-EVs in cancer treatment is in the area of photothermal therapy. It is well known that colloidal gold exhibits localized surface plasmon resonance that contributes to absorbing the light at a specific wavelength, resulting in photothermal properties and has been applied for hyperthermic cancer treatments^[53,54]^. Hence, the presence of GNPs in tt-Mfn-EVs offers a promising and safe therapeutic option in applying photothermal therapy for the treatment of localized cancers, especially those that are surgically inoperable. Thus, a combinatorial therapeutic modality of chemotherapeutic and photothermal therapeutic interventions could result in effective treatment outcomes. While tt-Mfn-EVs possess the essential hallmarks for developing EV-based cancer therapeutics, it is to be noted that the present study is limited to *in vitro* testing. However, prior to advancing the technology to clinical translation, it is important to test the stability, toxicity and efficacy of tt-Mfn-EVs in *in vivo* lung tumor models.

In a previous study from our laboratory, we tested TfR-targeted lipid nanoparticles (TfR-LNP) as a carrier for small interfering RNA (siRNA) delivery in *in vivo* lung tumor models^[37]^. In that study, we showed that systemic administration of TfR-siRNA LNP resulted in tumor-targeted selective delivery of therapeutic siRNA to subcutaneous and experimental lung metastasis resulting in effective tumor suppression and prolonged animal survival. The study showed transferrin ligand conjugated nanoparticles (TfNP) were safe and did not produce off-target non-specific toxicity. Additionally, administration of fluorescently labeled siRNA using TfNP showed gradual accumulation and retention of the siRNA-TfNP in the tumor with maximal accumulation observed at 24 h. In contrast, administration of non-targeted LNPs that did not have TfR-targeted ligand resulted in their accumulation in the liver and spleen, followed by rapid bioclearance. These findings, together with the results from the present study, underscore the importance of transferrin-receptor-targeted drug delivery for cancer therapy.

The limitations in the current study highlight two key areas for further development. First, EV yields from MRC-_9_ and other conventional cell lines are modest, and flask-based culture combined with downstream processing is both time-consuming and insufficient to produce the quantities required for rigorous animal studies and clinical translation. Identifying a cell source that robustly produces EVs at higher numbers and that is easy to grow on a large scale, and implementing scalable downstream platforms to ensure reproducible and high-yield EV production will overcome the limitation. Second, although our study results demonstrated selective tumor uptake and cytotoxicity, they are limited to *in vitro* models. It is therefore important to validate our study findings using appropriate *in vivo* tumor models for safety and efficacy.

Further, the limitations remain in establishing the suitability of using EVs as drug carriers over several liposome-based carriers is questioned frequently, in particular, when some of the liposome-based drug delivery (e.g., Doxil; Vyxeos) systems are already in use for cancer treatment, and testing of additional liposome-based drug delivery systems is in Phase I/II clinical testing for cancer treatment (www.clinicaltrials.gov). Our study showed that EVs were superior to liposomes in cell uptake, albeit limited to *in vitro* studies. The benefits of using EVs as drug carriers are that they possess all the properties akin to clinically approved synthetic liposome-based carriers and exceed in many ways, especially in being non-immunogenic and easy to manipulate. In addition, EVs can be isolated in bulk from natural sources (e.g., cells of human and plant origin) and lyophilized, which helps in their long-term storage and hence augments their feasibility over conventional liposomes. The challenge one can foresee is the scalability of tt-Mfn-EVs under good manufacturing practices (GMP) for use in humans. It is anticipated that advances in EV technologies in the future will enable us to overcome the scalability issues for clinical application of tt-Mfn-EVs. Overall, this study provides a platform for developing multifunctional EVs as drug carriers for cancer therapy.

In conclusion, we synthesized a tt-Mfn-EV as a drug carrier for cancer therapy and conducted studies using lung cancer as a model. Study results showed EVs could be decorated with tumor-targeting transferrin ligand and loaded with GNPs conjugated to CDDP via a pH-responsive linker for higher drug release under acidic conditions. The tt-Mfn-EV exhibited tumor-selective uptake and tumor cell killing with minimal cytotoxicity to normal MRC-_9_ and HEK_293_ cells. Additionally, testing of tt-Mfn-EV against HEK_293_ cells showed minimal toxicity, indicating the system could reduce CDDP-induced nephrotoxicity, which is a major treatment-related side effect observed in cancer patients. An added feature of our technology, although not tested, is the exploitation of the photothermal properties of the GNPs contained in the EVs for photodynamic therapy. This work offers a unique approach to combining nanotechnology with EV technology for developing innovative cancer therapeutics.

## DECLARATIONS

### Availability of data and materials

The authors confirm that the data presented in the article will be available from the corresponding author upon reasonable request.

### Copyright

© The Author(s) 2025.
