# Supplementary material for: Tumor-targeted multifunctional extracellular vesicles as drug carriers for lung cancer therapy
Source: Extracell Vesicles Circ Nucl Acids. 2025 Dec 24;6(4):1054–78. doi: 10.20517/evcna.2025.39 (PMC12809676; doi:10.20517/evcna.2025.39)
Supplement: Supplementary file 1 [file evcna-6-4-1054-SupplementaryMaterials.pdf]

Supplementary Materials

Tumor-targeted multifunctional extracellular vesicles as drug carrier for lung cancer therapy

Narsireddy Amreddy[1,2,†], Akhil Srivastava[1,2,‡], Natascha Riedinger[3], Murali Ragothaman[1,2], Yan D. Zhao[2,4], Hariprasad Gali[2,5], Anupama Munshi[2,6], Rajagopal Ramesh[1,2]

[1]Department of Pathology, University of Oklahoma Health Sciences Center, Oklahoma City, OK 73104, USA.
[2]OU Health Stephenson Cancer Center, University of Oklahoma Health Sciences Center, Oklahoma City, OK 73104, USA.
[3]Boone Pickens School of Geology, Oklahoma State University, Stillwater, OK 74078, USA.
[4]Department of Biostatistics and Epidemiology, University of Oklahoma Health Sciences Center, Oklahoma City, OK 73104, USA.
[5]Department of Pharmaceutical Sciences, University of Oklahoma Health Sciences Center, Oklahoma City, OK 73104, USA.
[6]Department of Radiation Oncology, University of Oklahoma Health Sciences Center, Oklahoma City, OK 73104, USA.
[†]Current Address: Cytovance Biologics, Oklahoma City, OK 73104, USA.
[‡]Current Address: Department of Pathology and Anatomical Sciences, University of Missouri School of Medicine, Columbia, MO 65212, USA.

Correspondence to: Dr. Rajagopal Ramesh, Department of Pathology, University of Oklahoma Health Sciences Center, Oklahoma City, OK 73104, USA. E-mail: rajagopal-ramesh@ou.edu

Results

GNP-CDDP synthesis. The gold nanoparticles surface was modified with PEG linkers to avoid the aggregations and for longer stability and enhanced permeability and retention effect[1]. The HS-PEG-OMe polymer with 8000MW was incubated with gold nanoparticles and the sulfohydryl (–SH) functional group was interacted with gold surface to form Au-S linkage. The outer functional group of methyl (–OMe) has inert propriety which could not react with other molecules. Further, the gold nanoparticles surface was modified with HS-PEG-COOH cross linker to conjugate with hydrated CDDP. Then the –COOH groups of Au-s-PEG-COOH complex was activated through isopropyl ethylamine base to initiate the reaction between –COOH and hydrated CDDP. The normal cisplatin was converted into hydrated CDDP in the presence of silver nitrate. The chlorine groups of cisplatin were replaced with –H_2O molecules. Estimation for hydrated CDDP by OPDA assay showed 74% of the yield obtained was hydrated. When the hydrated CDDP was incubated with activated Au-s-PEG-COOH complex, the pH sensitive coordinate ester linkage (-COO-Pt) was formed. The CDDP conjugation efficiency to the GNPs was estimated as 33.12% ± 3.04% by OPDA assay.

Fourier Transform Infrared (FTIR) results. The FTIR spectra represents the vibrational peaks of the functional groups. As shown in Supplementary Figure 1, the extracellular vesicles showed the peaks at 1640 cm^{-1} which attributed to the amide bond of C=O stretching vibrations, this amides might be contributing from extracellular vesicle membrane proteins and peptides[2,3]. The 1572 and 1543 cm^{-1} peaks corresponding to N-H bending vibrations of the proteins and peptides appeared with higher intensity. The -CH_2 antisymmetric and symmetric stretching vibrations appeared at 2918 and 2847 cm^{-1}, respectively. The membrane lipid acyl -CH_2 bending vibrations exhibited at 1472 and 1394 cm^{-1} with lower intensity. The GNP-CDDP vibrational spectra gives more intensity peak at 1081 cm^{-1}, which belongs to ester (O=C-O) bond in GNP-CDDP which arises from hydroxyl group in hydrated CDDP conjugated with activated carboxylate groups in GNP-PEG (GNP-PEG-COO^-) conjugations. This peak confirms the CDDP conjugated to GNPs through ester linkage. The other two major peaks appeared at 1635 and 1541 cm^{-1} represents the – NH_3 and CH_2 and bending vibrations of CDDP and PEG linkers. After forming the EV-GNP-CDDP complex, the major peak still appeared at 1081 cm^{-1} which belongs to O=C-O stretching vibrations

contributed in GNP-CDDP linkage, but peak intensity reduced when compared to GNP-CDDP peak and new peak appeared at 952 cm^{-1}[2]. The reduction in 1081 cm^{-1} peak indicates the Pt in O=C-O---Pt linkage might have interacted with extracellular vesicle membrane proteins that interaction resulted also new peak appeared at 952 cm^{-1}. This 952 cm^{-1} peak also might be corresponding to when extracellular vesicle membrane proteins interaction with Pt in CDDP. Another interesting observation made in the EV-GNP-CDDP spectra was that the extracellular vesicle membrane lipids, proteins and peptide peaks still appeared in EV-GNP-CDDP complex but with intensities drastically reduced indicating the extracellular vesicle surface was actively involved in EV-GNP-CDDP complex formation. The FTIR spectroscopy results concluded that CDDP was conjugated to GNPs through ester linkage and successfully loaded on to the extracellular vesicle membrane.

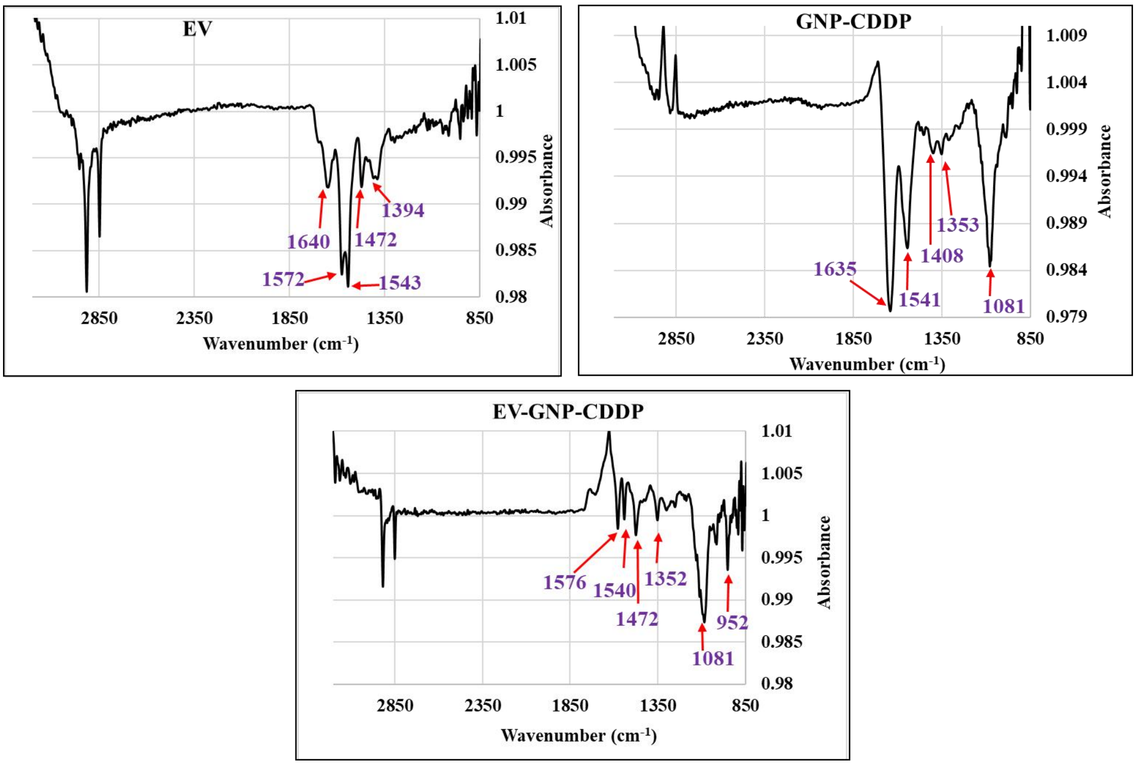

Supplementary Figure 1. FTIR spectroscopy analysis of extracellular vesicles, GNP-CDDP and EV-GNP-CDDP. FTIR spectra confirm characteristic functional groups and successful conjugation of GNP-CDDP to EVs.

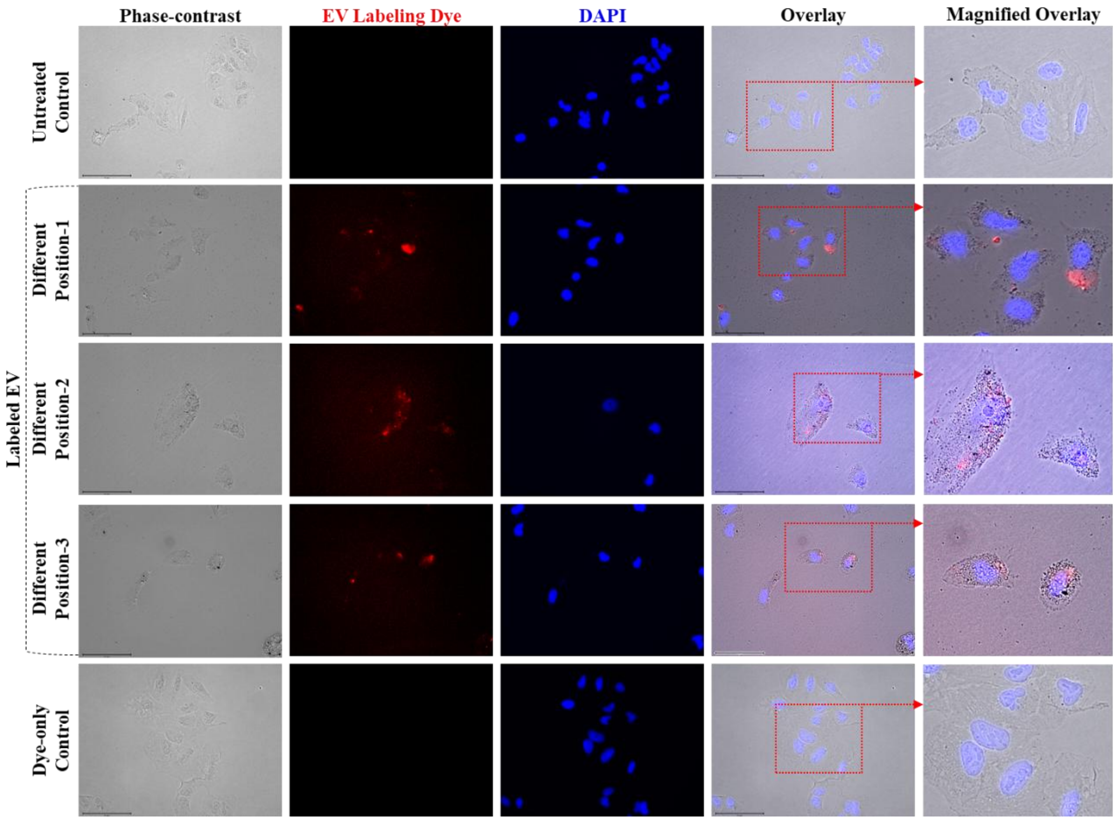

Supplementary Figure 2. Cellular uptake of fluorescently labeled EVs by A549 cells. EVs that were fluorescently labeled using the ExoGlow-Protein kit were added to A549 cells. Cells that were treated with unlabeled EVs and fluorescent dye only served as controls. EVs uptake by cells were captured by fluorescence microscopy at 24 h post-treatment. Red fluorescence indicates labeled EVs; blue fluorescence (DAPI) marks cell nuclei. A549 cells treated with labeled EV exhibited distinct red fluorescence, whereas dye-only controls showed no signal, confirming EV-specific uptake. These findings validate the specificity of the labeling strategy and highlight the role of transferrin-functionalization in enhancing cellular internalization and therapeutic efficacy. Magnification: 40×; scale bar: 75 µm.

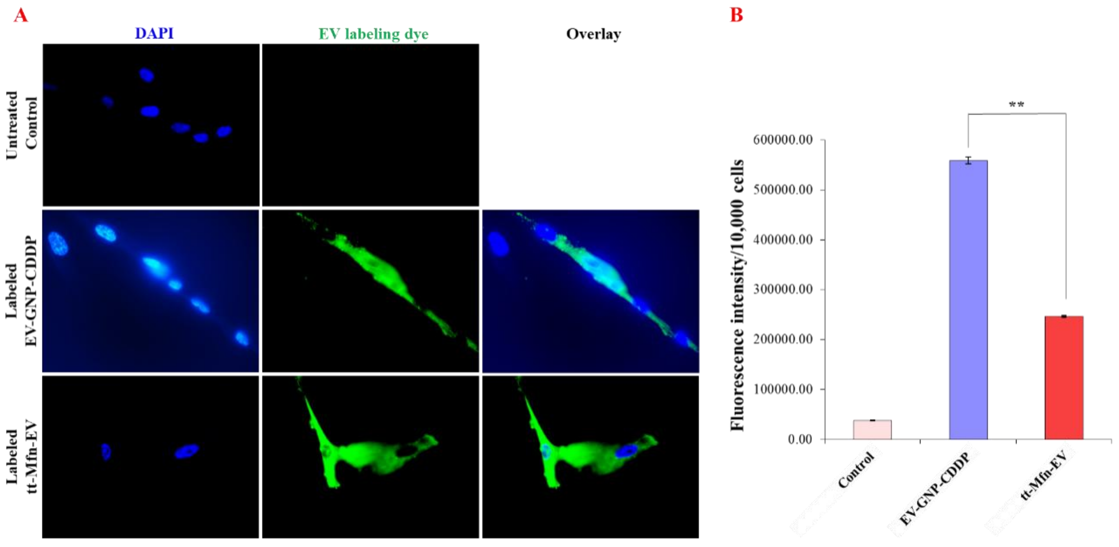

Supplementary Figure 3. Cell uptake of labeled EV-GNP-CDDP and tt-Mfn-EV in MRC-9 cells. (A) Fluorescence microscopy images of MRC-9 cells at 24 h after treatment with fluorescently labeled EV-GNP-CDDP and tt-Mfn-EVs. (B) Quantification of cellular uptake was performed by measuring fluorescence intensity in cells treated with fluorescently labeled EV-GNP-CDDP and tt-Mfn-EV, compared to untreated controls. Cell uptake of EVs was observed in both fluorescently labeled EV-GNP-CDDP and tt-Mfn-EV with greatest uptake observed in EV-GNP-CDDP-treated cells. Asterisk denotes significance, $^{**}P < 0.01$.

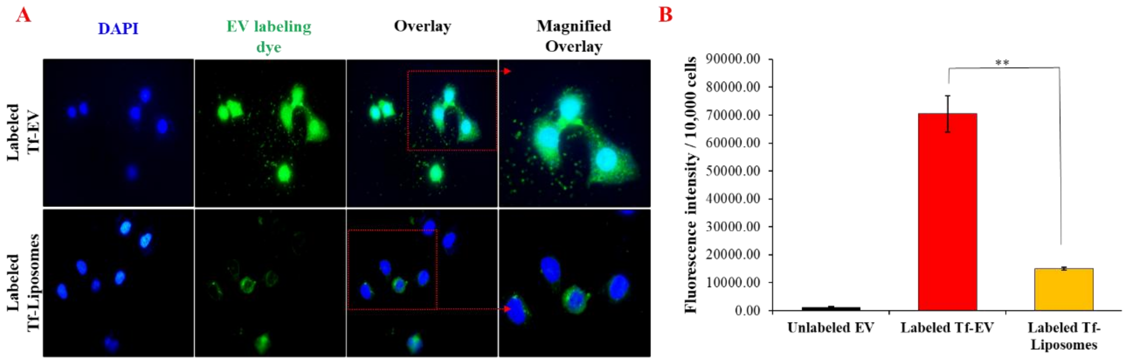

Supplementary Figure 4. Comparison of transferrin (Tf) conjugated EVs and liposomes uptake by A549 tumor cells. Cells treated with fluorescently labeled Tf-liposomes and Tf-EVs were assessed for cell uptake by (A) fluorescence imaging, and (B) by measuring fluorescence intensity. Cells treated with unlabeled EVs served as control. Cell uptake of Tf-EVs was significantly higher compared to Tf-liposomes indicating EVs are more efficiently taken up by tumor cells than liposomes. Asterisk denotes significance, $^{**}P < 0.01$.

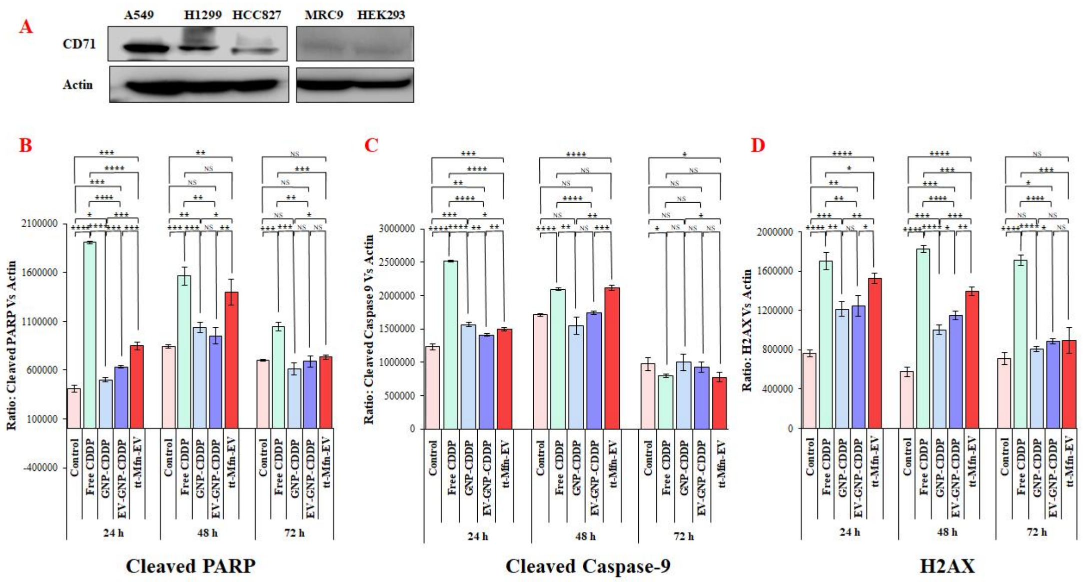

Supplementary Figure 5. Transferrin receptor expression in lung cancer and normal cells, and quantification of apoptotic and DNA damage markers in A_{549} cells post-treatment. (A) Western blot analysis reveals the elevated transferrin receptor expression in lung cancer (A_{549}, H_{1299}, HCC_{827}) cells relative to normal (MRC-$_{9}$ and HEK_{293}) cells. (B-D) Quantification graphs of corresponding western blot analysis of cleaved PARP, cleaved caspase $_{9}$ and H_2AX normalized against actin in A_{549} lung cancer cells receiving various treatments. Asterisk denotes significance, $^{*}P < 0.05$; $^{**}P < 0.01$; $^{***}P < 0.001$; $^{****}P < 0.0001$; NS denotes, not significant.

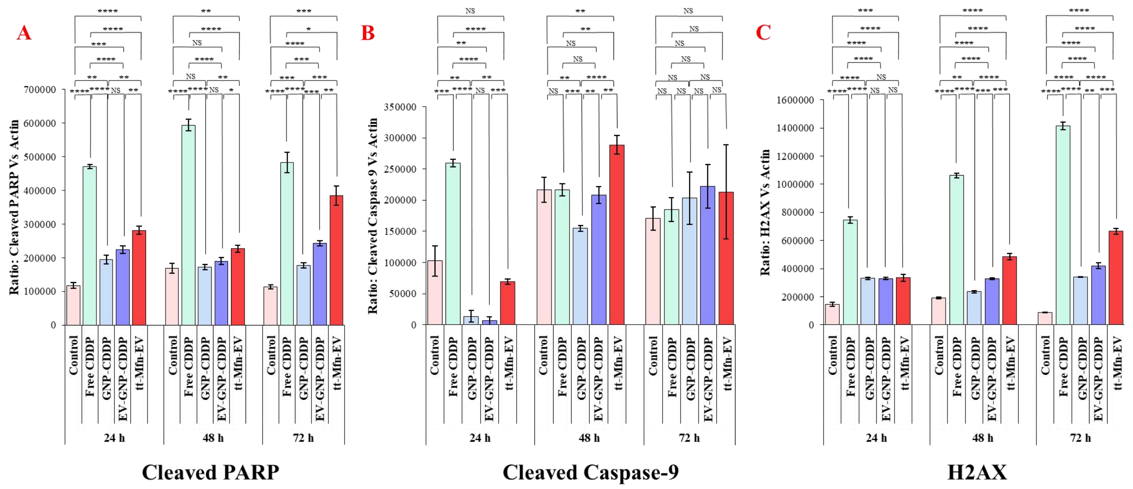

Supplementary Figure 6. Apoptotic and DNA damage markers quantification in HCC_{827} cells post-treatment. Quantification graphs of corresponding western blot analysis of (A) cleaved PARP, (B) cleaved caspase $_9$ and (C) H_2AX normalized against actin in HCC_{827} lung cancer cells receiving various treatments. Asterisk denotes significance, $^{*}P < 0.05$; $^{**}P < 0.01$; $^{***}P < 0.001$; $^{****}P < 0.0001$; NS denotes, not significant.

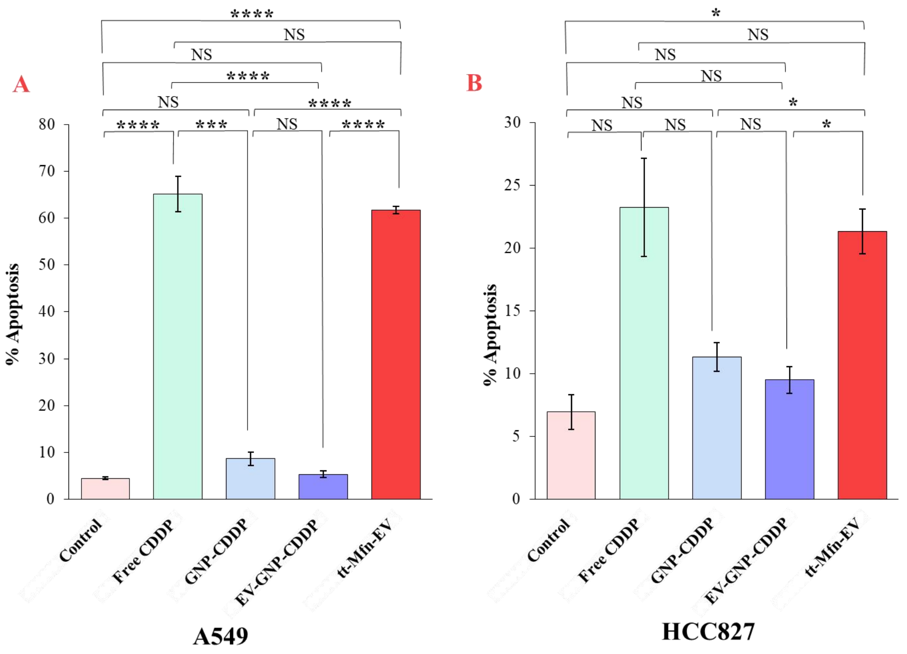

Supplementary Figure 7. Analysis of tumor cell apoptosis by AnnexinV/PI staining in A549 and HCC827 cells. (A) A549 and (B) HCC827 lung tumor cells were treated with free CDDP, GNP-CDDP, EV-GNP-CDDP and tt-Mfn-EV and analyzed for apoptotic cells at 24 h after treatment. Untreated cells served as controls. Asterisk denotes significance, $^{*}P < 0.05$; $^{***}P < 0.001$; $^{****}P < 0.0001$; NS denotes, not significant.

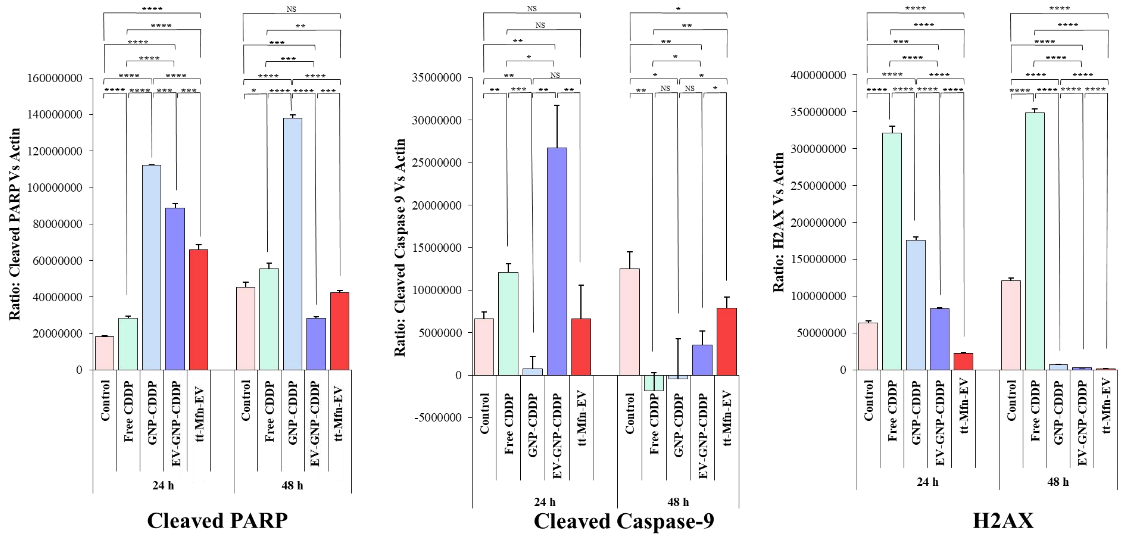

Supplementary Figure 8. Apoptotic and DNA damage markers quantification in MRC-9 cells. Quantification graphs of corresponding western blot analysis of cleaved PARP, cleaved caspase 9 and H_2AX normalized against actin in normal human lung fibroblast MRC-9 cell line receiving various treatments. Asterisk denotes significance, $^{*}P < 0.05$; $^{**}P < 0.01$; $^{***}P < 0.001$; $^{****}P < 0.0001$; NS denotes, not significant.

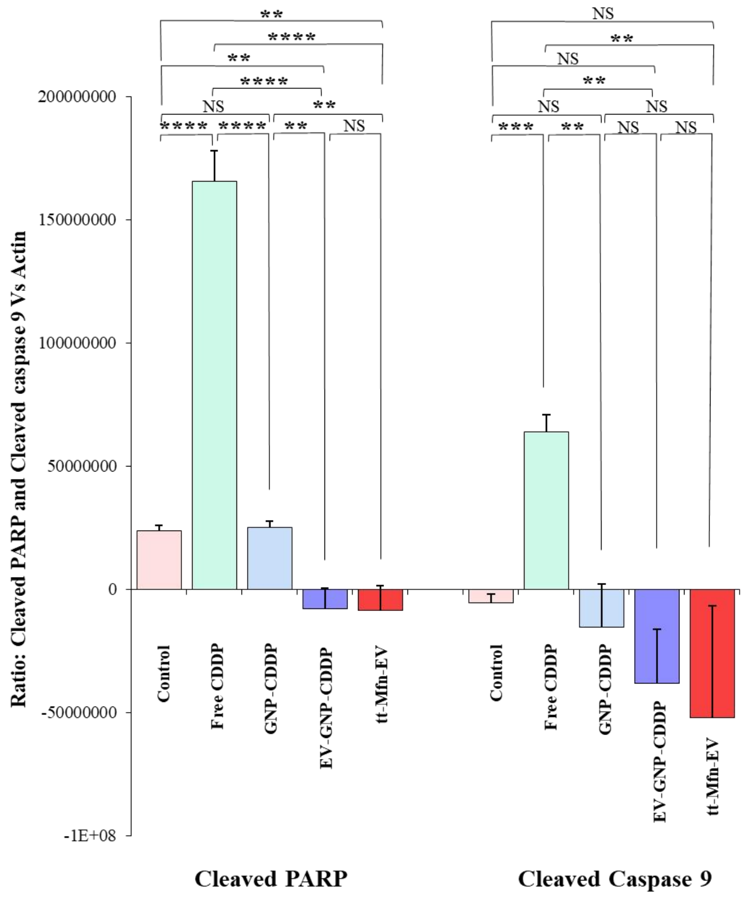

Supplementary Figure 9. Apoptotic markers quantification in HEK_{293} cells. Quantification graphs of corresponding western blot analysis of cleaved PARP and cleaved caspase $_9$ normalized against actin in HEK_{293} kidney cells receiving various treatments. Asterisk denotes significance, $^{**}P < 0.01$; $^{***}P < 0.001$; $^{****}P < 0.0001$; NS denotes, not significant.

References

1. Hagiwara K, Kurihara K, Honma M, Yamamoto J, Shinohara F. PEG-modification on the endo-position of an antisense oligonucleotide increases tumor accumulation via the EPR effect. *J. Biomater. Sci. Polym. Ed.* 2018;29:448-59. [PMID: 29318941 DOI: https://doi.org/10.1080/09205063.2017.1422853.]
2. Nguyen NT, Nguyen NNT, Tran NTN, Le PN, Nguyen TBT, et al. Synergic Activity Against MCF-7 Breast Cancer Cell Growth of Nanocurcumin-Encapsulated and Cisplatin-Complexed Nanogels. *Molecules* 2018;23:3347. [PMID: 30567316 DOI: https://doi.org/10.3390/molecules23123347.]
3. Zlotogorski-Hurvitz A, Dekel BZ, Malonek D, Yahalom R, Vered M. FTIR-based spectrum of salivary exosomes coupled with computational-aided discriminating analysis in the diagnosis of oral cancer. *J. Cancer Res. Clin. Oncol.* 2019;145:685-94. [PMID: 30603907 DOI: https://doi.org/10.1007/s00432-018-02827-6.]
